# Actinide–Pnictide (An−Pn) Bonds Spanning Non‐Metal, Metalloid, and Metal Combinations (An=U, Th; Pn=P, As, Sb, Bi)

**DOI:** 10.1002/anie.201711824

**Published:** 2017-12-29

**Authors:** Thomas M. Rookes, Elizabeth P. Wildman, Gábor Balázs, Benedict M. Gardner, Ashley J. Wooles, Matthew Gregson, Floriana Tuna, Manfred Scheer, Stephen T. Liddle

**Affiliations:** ^1^ School of Chemistry The University of Manchester Oxford Road Manchester M13 9PL UK; ^2^ Institute of Inorganic Chemistry University of Regensburg Universitätsstr.31 93053 Regensburg Germany

**Keywords:** metalloids, metal–metal bonds, pnictides, thorium, uranium

## Abstract

The synthesis and characterisation is presented of the compounds [An(Tren^DMBS^){Pn(SiMe_3_)_2_}] and [An(Tren^TIPS^){Pn(SiMe_3_)_2_}] [Tren^DMBS^=N(CH_2_CH_2_NSiMe_2_Bu^t^)_3_, An=U, Pn=P, As, Sb, Bi; An=Th, Pn=P, As; Tren^TIPS^=N(CH_2_CH_2_NSiPr^i^_3_)_3_, An=U, Pn=P, As, Sb; An=Th, Pn=P, As, Sb]. The U−Sb and Th−Sb moieties are unprecedented examples of any kind of An−Sb molecular bond, and the U−Bi bond is the first two‐centre‐two‐electron (2c–2e) one. The Th−Bi combination was too unstable to isolate, underscoring the fragility of these linkages. However, the U−Bi complex is the heaviest 2c–2e pairing of two elements involving an actinide on a macroscopic scale under ambient conditions, and this is exceeded only by An−An pairings prepared under cryogenic matrix isolation conditions. Thermolysis and photolysis experiments suggest that the U−Pn bonds degrade by homolytic bond cleavage, whereas the more redox‐robust thorium compounds engage in an acid–base/dehydrocoupling route.

The preparation, isolation, and study of new molecular element–element bonds remains a fundamentally important endeavour because it informs us about chemical characteristics and reactivity, allows us to probe and refine periodic trends, and provides vital benchmarking for structural and theoretical predictions and modelling. Reflecting much progress over decades of synthetic effort, there are now few places left in the Periodic Table where new element–element bonds can be regularly discovered. However, the actinides, where progress has generally lagged owing to their radioactivity and the inherent challenges involved in stabilizing chemical bonds to some of the largest metal ions in existence, remains a rich seam from which to mine new chemical bonds. For example, only very recently have the first Am−S, Pu−C, and Bk−O bonds been crystallographically authenticated.^1^

The aforementioned examples all involve synthetic transuranic examples with unique associated challenges; studies have in particular been impeded by their radioactive nature, need for specialist handling facilities, and limited availabilities. However, even for naturally occurring neighbour elements like uranium and thorium, which can be handled in normal laboratories, there are chemical bond combinations yet to be realised. For example, regarding An−Pn bonds (An=U, Th; Pn=N, P, As, Sb, Bi), although covalent An−N, An−P, and An−As bonds are known,^2^, ^3^, ^4^ somewhat remarkably given the burgeoning nature of non‐aqueous actinide chemistry,^5^ An−Sb and An−Bi derivatives are conspicuous by their absence even though analogous examples are known in transition‐metal^6^ and even in lanthanide chemistry.^7^ Indeed, there are no structurally characterized U−Sb, Th−Sb, or Th−Bi bonds and there is only one report of U−Bi bonds,^8^ which involves open‐shell, delocalised radical Zintl clusters that reside at the molecular–periodic interface. Seeking to remedy this situation, we sought to extend our previous work on early metal−PnH_2_ complexes.3c, 4, 9 Since discrete (PnH_2_)^−^ anions are not available for Sb and Bi, and noting that many heavy PnH_*x*_R_3−*x*_ reagents are prone to facile Pn−C bond homolysis, we utilised the more sterically demanding pnictides {Pn(SiMe_3_)_2_}^−^ for P, As, Sb, and Bi, though even these reagents are prone to easy decomposition.^10^, ^11^ We reasoned that this would present the opportunity to prepare a structurally homologous series of An−Pn covalent bond benchmarks, whilst enabling meaningful comparison of the Pn geometries (that is, development of trigonal pyramidal from trigonal planar) as the pnictide group is descended.

Herein, we report the synthesis and characterisation of molecular compounds containing new An−Pn bonds that include the first structurally authenticated U−Sb and Th−Sb bonds of any kind and the first two‐centre‐two‐electron (2c–2e) U−Bi bond. The corresponding Th−Bi bond was too unstable to isolate, highlighting the major challenges of preparing these ill‐suited hard–soft linkages generally. These complexes present chemical bond benchmarks, and the U−Bi bond is the heaviest 2c–2e pairing of two elements involving an actinide under macroscopic, ambient conditions, exceeded experimentally only by An−An pairings in matrix isolation experiments.^12^ Preparing homologues spanning non‐metal, metalloid, and metal within a single element group has permitted elucidation of a formal periodic break‐point; DFT calculations suggest that P and As adopt formal −3 oxidation states whereas Sb and Bi are more appropriately assigned as +1.

To assemble the desired An−Pn linkages we utilised a salt elimination strategy with separated ion pair An precursors to avoid complications with installation of soft Pn centres at hard An ions since the outer‐sphere borate is an excellent leaving group.3c, 4, 9 Thus, treatment of [An(Tren^DMBS^)(L)][BPh_4_] (An=U, L=THF, **1U**; An=Th, L=DME, **1Th**) or [An(Tren^TIPS^)(L)][BPh_4_] (An=U, L=THF, **2U**; An=Th, L=DME, **2Th**)3c, 4, 9 with KPn(SiMe_3_)_2_ (Pn=P, As, Sb, Bi)^10^ afforded [An(Tren^DMBS^){Pn(SiMe_3_)_2_}] (**3AnPn**) and [An(Tren^TIPS^){Pn(SiMe_3_)_2_}] (**4AnPn**).^11^ Most combinations proved accessible, and **3UP**, **3UAs**, **3USb**, **3UBi**, **3ThP**, **3ThAs**, **4UP**, **4UAs**, **4USb**, **4ThP**, **4ThAs**, and **4ThSb** were isolable (Scheme 1). For completeness, we examined installation of the analogous amide {N(SiMe_3_)_2_}^−^, but found only the formation of Tren‐cyclometallates, which seems to be sterically driven, since for example the dicyclohexylamide complex [U(Tren^DMBS^){N(C_6_H_11_)_2_}] is isolable.^13^ The characterisation data for the isolable complexes are consistent with their formulations, and for uranium the variable temperature magnetisation data (Supporting Information, Figures S1–S7)^11^ corroborate the uranium(IV) assignments, but otherwise are not particularly informative so we determined their molecular structures to gain further insight.

**Scheme 1:**
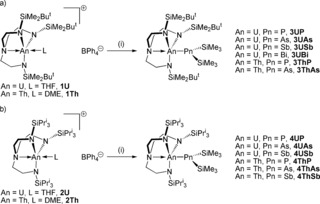
Synthesis of the An−Pn complexes reported in this study: a) utilising the triamidoamine Tren^DMBS^ ancillary ligand; b) utilising the triamidoamine Tren^TIPS^ ancillary ligand. Reagents and conditions for (a) and (b): i) THF, KPn(SiMe_3_)_2_, −78 °C to room temperature.

The solid‐state molecular structures of **3UP**, **3UAs**, **3USb**, **3UBi**, **3ThP**, **3ThAs**, **4UP**, **4UAs**, **4USb**, **4ThP**, **4ThAs**, and **4ThSb** were all determined by single‐crystal X‐ray diffraction. The structures of **3UP**, **3UAs**, **3USb**, and **3UBi** are illustrated in Figure 1 and key metrical parameters are compiled in Table 1. The other structurally determined compounds in this study are in the Supporting Information, Figures S10–S20.^11^ For the sake of brevity our discussion will largely focus on the **3UPn** series since this constitutes a complete actinide–heavy‐pnictide family.

**Figure 1:**
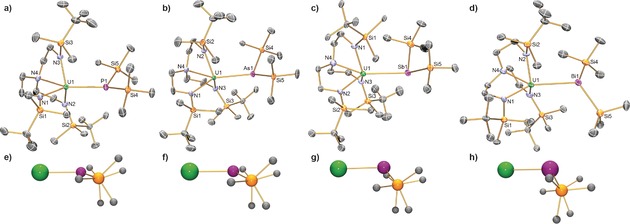
a)–d) Solid‐state molecular crystal structures of **3UP**, **3UAs**, **3USb**, and **3UBi**, respectively, measured at 120 K. Ellipsoids set at 40 % probability; hydrogen atoms, minor disorder components, and any lattice solvent removed for clarity.^23^ e)–h) Ball‐and‐stick representations of the core U‐Pn(SiMe_3_)_2_ units from the same side‐on perspective in each case, where one Si perfectly obscures the other, to show the increasing deviation from trigonal planar to trigonal pyramidal geometry as the pnictide series is descended; all other atoms in these depictions are omitted for clarity. U green, Pn magenta, N blue, Si orange, C gray.

**Table 1:** An−Pn bond lengths [Å] and sum of Pn angles [°] for the structurally authenticated molecules in this study.

Combination	**3UPn**	**4UPn**	**3ThPn**	**4ThPn**
Pn=P	2.8646(14)/ 354.06(8)	2.8391(9)/ 359.94(5)	2.9406(11)/ 351.28(7)	2.9020(13)/ 356.75(8)
Pn=As	2.9423(9)/ 349.71(9)	2.9062(7)/ 355.56(8)	3.0456(9)/ 343.47(9)	2.9569(6)/ 359.35(6)
Pn=Sb	3.2437(8)/ 325.96(11)	3.2089(6)/ 351.53(12)	–	3.2849(3)/ 348.13(4)
Pn=Bi	3.3208(4)/ 315.98(9)	–	–	–

The U−Pn distances of 2.8646(14), 2.9423(9), 3.2437(8), and 3.3208(4) Å for **3UP**, **3UAs**, **3USb**, **3UBi**, respectively, can be compared to the respective sums of single‐bond covalent radii of 2.81, 2.91, 3.10, and 3.21 Å,^14^ and for **3UP** to the U−P distance of 2.789(4) Å in sterically less encumbered [U(η^5^‐C_5_Me_5_)_2_(Cl){P(SiMe_3_)_2_}];3h these data essentially divide into two groups where the experimental U−P and U−As pairs are within 0.05 Å of those predicted, but the discrepancies for the U−Sb and U−Bi distances are >0.1 Å. This suggests a periodic break between As and Sb, but also perhaps reflects the changing sum of angles at each Pn (P, 354.06(8); As, 349.71(9); Sb, 325.96(11); Bi, 315.98(9)°); although respective s–p energy gaps decrease as Group 15 is descended, rehybridisation promotion energies conversely increase owing to progressively inefficient s–p orbital overlap from increasingly diffuse orbitals.

Interestingly, for **3ThP** and **3ThAs** the Th−Pn distances are 0.08–0.1 Å longer than the uranium analogues even though the single bond covalent radius of Th is only 0.05 Å larger than that of U.^14^ Conversely, however, the U−Pn distances for **4UP**, **4UAs**, and **4USb**, are all shorter than the respective corresponding U−Pn distances in the **3UPn** series. Also, the sum of angles at Pn varies far less for the **4UPn** than **3UPn** series, which can be related to the sterically more demanding nature of Tren^TIPS^ compared to Tren^DMBS^ restricting the tendency towards orthogonal bonding for the heavier pnictides. This suggests that the trigonal planar bonding mode of these pnictides is stronger than a more trigonal pyramidal mode. This trend is overall repeated when comparing the **3ThPn** and **4ThPn** series together, though we note that the differences for **4ThPn** vs. **4UPn** are smaller than those of **3ThPn** vs. **3UPn**, reflecting the greater constraints imposed by the more sterically demanding Tren^TIPS^ compared to Tren^DMBS^.

The UV/Vis/NIR spectroscopic data for **3UPn** (Supporting Information, Figure S8)^11^ show a characteristic absorption maxima that bathochromically shifts (**3UP**, 19,420; **UAs**, 18,280; **USb**, 15,820; **UBi** 14,245 cm^−1^); this can be related to the increasing pyramidalisation of the Pn centres as the Pn group is descended and a decreasing pnictide–uranium charge transfer energy. The same trend is observed for **4UPn** (Supporting Information, Figure S9).^11^

To further probe the An−Pn linkages, we examined them using DFT, NBO, and QTAIM methods (Table 2; Supporting Information, Figures S21–S32).^11^ Computed structures compare well with the solid‐state structures, so we conclude that these models represent qualitative pictures of the electronic structures of these complexes. The An−Pn Mayer bond orders, considering these linkages are expected to be polar, are surprisingly high, suggest Pn π‐donation in addition to the anticipated σ‐bonds.^15^

**Table 2:** Selected computed DFT, NBO, and QTAIM data.

	Bond length and index^[b,c]^		Charges^[d]^		Spin density^[e]^	NBO σ‐component^[g]^		NBO π‐component^[g]^			QTAIM^[h]^			
Entry^[a]^	An−Pn	BI	*q*_An_	*q*_Pn_	*m*_An_	An[%]	Pn[%]	An[%]	Pn[%]	An 7s/7p/6d/5f	*ρ*(r)	∇^2^ *ρ*(r)	*H*(r)	*ϵ(r)*
**3UP**	2.898	0.92	2.24	−1.02	2.33	0	100	14	87	1:1:16:82	0.03	0.04	−0.01	0.27
**3UAs**	2.988	0.94	2.33	−1.32	2.37	0	100	17	83	1:1:17:81	0.02	0.03	−0.01	0.29
**3USb**	3.265	0.87	1.85	−0.39	2.38	0	100	17	83	5:1:19:75	0.02	0.02	−0.01	0.29
**3UBi**	3.385	0.78	1.86	−0.40	2.38	0	100	18	82	9:2:22:67	0.02	0.01	−0.01	0.15
**4UP**	2.854	1.00	2.43	−0.97	2.37	0	100	14	86	0:1:29:70	0.03	0.04	−0.01	0.22
**4UAs**	2.944	0.98	2.69	−1.31	2.39	0	100	17	83	0:1:29:70	0.02	0.03	−0.01	0.26
**4USb**	3.222	1.03	2.13	−0.33	2.48	0	100	8	92	36:1:14:49	0.02	0.03	−0.01	0.13
**3ThP**	2.981	0.81	2.17	−1.05	–	0	100	6	94	2:1:23:74	0.04	0.06	−0.01	0.22
**3ThAs**	3.072	0.86	2.29	−1.32	–	0	100	6	94	6:1:32:61	0.04	0.05	−0.01	0.26
**4ThP**	2.955	0.87	2.44	−0.99	–	0	100	5	95	17:1:33:49	0.04	0.07	−0.01	0.31
**4ThAs**	2.996	0.94	2.58	−1.33	–	0	100	6	94	0:2:36:62	0.04	0.07	−0.01	0.37
**4ThSb**	3.315	0.92	2.14	−0.36	–	0	100	7	93	19:2:36:43	0.03	0.05	−0.01	0.25

[a] All molecules geometry optimised without symmetry constraints using the BP86 GGA functional and a basis set derived from TZP/ZORA all‐electron ADF database; calculations were unrestricted for uranium and restricted for thorium. [b] Computational An−Pn distances [Å]. [c] Mayer bond indices. [d] MDC‐q charges on An. [e] MDC‐m α‐spin densities on An. [f] MDC‐q charges on Pn. [g] Natural bond orbital (NBO) analyses; the electron occupancies of these orbitals are ≥97 %. [h] QTAIM topological electron density [*ρ*(r)], Laplacian [∇^2^ *ρ*(r)], electronic energy density [*H*(r)], and ellipticity [*ϵ(r)*] bond critical point data.

The computed An charges, and spin densities for uranium, are overall consistent with their +4 oxidation states,2a but the computed charges of the Pn centres fall into two clear groups; for P/As computed charges are about −1 to −1.3 whereas for Sb/Bi they are lower at about −0.3 to −0.4 and this does not appear to be related in any way to the geometry of the Pn centre as an explanation. This suggests a periodic break where the former pair are best described formally as being in the −3 oxidation state whereas the latter two are better formulated as being +1.

When the Pn centre remains essentially trigonal planar, as is the case for the **4UPn** and **4ThPn** series, the An charge follows the trend An−As > An−P > An−Sb, which suggests that As is the weakest donor ion. Interestingly, for the **3UPn** series the An charges increases from **3UP** to **3UAs**, but then falls away for **3USb** and **3UBi**, so that the same, but extended, series of An charges of An−As > An−P ≫ An−Sb ≈An−Bi emerges. This is counterintuitive, because the expected trend would be for the An charges to be ordered An−Bi > An−Sb > An−As > An−P as suggested by the Mayer bond order data. However, inspection of the DFT Kohn Sham and NBO descriptions of **3UP**, **3UAs**, **3USb**, **3UBi**, **3ThP**, **3ThAs**, **4UP**, **4UAs**, **4USb**, **4ThP**, **4ThAs**, and **4ThSb** reveals that whilst the An−Pn σ‐bonds are largely ionic, surprisingly, since the Pn *n*p‐orbitals (*n=*3–6) become increasingly diffuse, there are significant π‐bonding combinations in these complexes, and, using series **3UPn**, as the pnictide becomes more pyramidalised although one lobe of the p‐orbital moves increasingly away from the metal the other lobe approaches much more closely and so may actually engage more effectively overall with one orbital lobe than two. Thus, linkages that would be expected to be weaker may actually be stronger, with respect to donor strength, which in this context is not synonymous with thermodynamic enthalpic bond strength.

The asymmetry of the An−Pn bonding, as suggested by DFT and NBO methods, is further supported by analysis of the QTAIM data; although highly polar bonds are certainly found, the bond critical point ellipticity values are consistently greater than zero and of the magnitude found for the C−C bonds in benzene tending to ethene.^16^

Although **4UBi**, **3ThSb**, **3ThBi**, and **4ThBi** have eluded isolation, attempts to prepare them along with studies on the subsequent reactivity of the isolable An−Pn complexes has proven informative with respect to unravelling the underpinning chemistry of these An−Pn linkages. Attempts to prepare **4UBi** from **2U** and KBi(SiMe_3_)_2_ resulted in batch‐variable quantities of green crystals, that could not be cleanly isolated, of (Me_3_Si)_2_Bi−Bi(SiMe_3_)_2_ (**Bi_2_**)^11^, ^17^ as verified by single‐crystal X‐ray diffraction. This implies that **4UBi** is transiently formed, but decomposes by homolytic U−Pn bond cleavage to give [U(Tren^TIPS^)],^18^ which was indeed identified in reaction mixtures by ^1^H NMR spectroscopy. Interestingly, attempts to prepare **3ThSb**, **3ThBi**, and **4ThBi** resulted in the isolation, respectively, of variable levels of red (Me_3_Si)_2_Sb−Sb(SiMe_3_)_2_ (**Sb_2_**),^11^, ^19^ and green **Bi_2_**, verified by single‐crystal X‐ray diffraction. Since the Th^4+^/Th^3+^ reduction potential is very negative,^20^ homolytic Th−Pn bond cleavage seems unlikely, but on one occasion, from otherwise intractable, complex reaction mixtures, crystals of the cyclometallate complex [Th{N(CH_2_CH_2_NSiMe_2_Bu^t^)_2_(CH_2_CH_2_NSiMeBu^t^CH_2_)}(DME)] (**5**) were isolated from an attempted preparation of **3ThSb**.^11^ This suggests that a concerted or step‐wise deprotonation/cyclometallation–dehydrocoupling of HPn(SiMe_3_)_2_ reaction occurs for thorium.

To probe the mechanistic aspects further we investigated thermal and photolytic U−Pn reactivity profiles; analogous thorium studies gave no different outcomes to the ones described above. Complex **4USb**, and putative **4UBi** prepared in situ, completely decompose at 80 °C to give [U(Tren^TIPS^)] and **Sb_2_** or **Bi_2_**, respectively, consistent with homolytic bond cleavage. Under these conditions, **4UP** and **4UAs** and surprisingly **3USb** and **3UBi** show little decomposition (<5 %). Photolysis (125 W UV lamp, 2 h) of **4USb** and putative **4UBi** prepared in situ results in conversion into the cyclometallate complex [U{N(CH_2_CH_2_NSiPr^i^_3_)_2_(CH_2_CH_2_NSiPr^i^_2_CHMeCH_2_)}]^21^ and elemental Sb or Bi. Interestingly, photolysis of **3USb** and **3UBi** results in initial formation of **Sb_2_** or **Bi_2_**, H_2_, and the cyclometallate [U{N(CH_2_CH_2_NSiMe_2_Bu^t^)_2_(CH_2_CH_2_NSiMeBu^t^CH_2_)}],^22^ but **3UP** and **3UAs** show little decomposition under photolytic conditions. Extended photolysis of **3USb** and **3UBi** resulted in **Sb_2_**/**Bi_2_** decomposition to elemental Sb/Bi, which was verified by independent decomposition of **Sb_2_**/**Bi_2_** under the same conditions. Under photolytic conditions, [U(Tren^R^)] species slowly cyclometallate with elimination of H_2_. So, the above data suggest that for U−Pn bonds homolysis is the preferred decomposition route, which may proceed to production of uranium–cyclometallate and elemental pnictide deposition under photolytic but not thermal conditions, but the Th−Pn linkages undergo acid–base/dehydrocoupling reactions owing to the redox robustness of thorium. The more facile decomposition of **4USb** and “**4UBi”** compared to **3USb** and **3UBi** suggest that although sterically demanding ligands are necessary to stabilise these polar U−Pn linkages that Tren^TIPS^ may be too bulky and actually destabilise the U−Sb/U−Bi linkages; the isolation of **3UBi** is thus remarkable because this complex has the facile uranium redox bond homolysis route open to it yet it is still isolable. This is clearly a delicate balance of sterics, since for the larger thorium neither Tren^DMBS^ nor Tren^TIPS^ can stabilise Th−Bi linkages. Such a fine balance of metal/ligand size ratio has been found previously with respect to μ‐phosphido linkages where thorium–Tren^TIPS^ gives stable ThPTh linkages but uranium–Tren^TIPS^ results in UPU linkages that readily decompose.9a,9d

To conclude, we have reported the synthesis and characterisation of new An−Pn bonds that include the first structurally authenticated U−Sb and Th−Sb bonds of any kind and the first 2c–2e U−Bi bond. The corresponding Th−Bi bond was too unstable to isolate, highlighting the major challenges of preparing these mismatched hard–soft linkages generally. These complexes present chemical bond benchmarks, and the U−Bi bond is the heaviest 2c–2e pairing of two elements involving an actinide under macroscopic, ambient conditions, exceeded only by An−An pairings in matrix isolation experiments. Preparing homologues spanning non‐metal, metalloid, and metal within a single element group has permitted elucidation of a formal periodic break‐point between As and Sb. Reactivity studies suggest that the heavier U−Pn bonds decompose by homolytic U−Pn bond cleavage, and the resulting uranium(III) and di‐pnictane compounds react further to give uranium(IV)–cyclometallate, hydrogen, and elemental pnictide, respectively, whereas the more redox robust thorium complexes engage in an acid–base/dehydrocoupling route to give thorium–cyclometallate and di‐pnictane. Thus, although the same product classes emerge from these decomposition reactions overall they proceed via different mechanistic routes, highlighting the different redox chemistries of uranium and thorium actinide elements.

*Dedicated to Professor William J. Evans on the occasion of his 70th birthday*

## Conflict of interest

The authors declare no conflict of interest.

## Supporting information

As a service to our authors and readers, this journal provides supporting information supplied by the authors. Such materials are peer reviewed and may be re‐organized for online delivery, but are not copy‐edited or typeset. Technical support issues arising from supporting information (other than missing files) should be addressed to the authors.

Supplementary
