# Supplementary material for: Actinide–Pnictide (An−Pn) Bonds Spanning Non‐Metal, Metalloid, and Metal Combinations (An=U, Th; Pn=P, As, Sb, Bi)
Source: Angew Chem Int Ed Engl. 2017 Dec 29;57(5):1332–6. doi: 10.1002/anie.201711824 (PMC5814731; doi:10.1002/anie.201711824)
Supplement: Supplementary file 1 — Supplementary [file ANIE-57-1332-s001.pdf]

Angewandte Chemie
Eine Zeitschrift der Gesellschaft Deutscher Chemiker

Supporting Information

Actinide–Pnictide (An−Pn) Bonds Spanning Non-Metal, Metalloid, and Metal Combinations (An = U, Th; Pn = P, As, Sb, Bi)

Thomas M. Rookes[+], Elizabeth P. Wildman[+], Gábor Balázs, Benedict M. Gardner, Ashley J. Wooles, Matthew Gregson, Floriana Tuna, Manfred Scheer, and Stephen T. Liddle**

anie_201711824_sm_miscellaneous_information.pdf

Experimental

General

All manipulations were carried out using Schlenk techniques, or an MBraun UniLab glovebox, under an atmosphere of dry nitrogen. Solvents were dried by passage through activated alumina towers and degassed before use or were distilled from calcium hydride. All solvents were stored over potassium mirrors except ethers which were stored over activated 4 Å sieves. Deuterated solvent was distilled from potassium, degassed by three freeze-pump-thaw cycles and stored under nitrogen. $[U(Tren^{R})(THF)][BPh_4]$ ($Tren^{R}$ = $Tren^{TIPS}$: $\{N(CH_2CH_2NSiPr^{i}_3)_3\}$ or $Tren^{DMBS}$: $\{N(CH_2CH_2NSiMe_2Bu^{t})_3\}$) and $[Th(Tren^{TIPS})(DME)][BPh_4]$ were prepared as described previously.[1-3] $KPn(SiMe_3)_2$) (E = P, As, Sb) were prepared through modification of published procedures.[4-6]

^{1}H, ^{13}C, ^{29}Si, and ^{31}P NMR spectra were recorded on a Bruker 400 spectrometer operating at 400.2, 100.6, 79.5, and 162.0 MHz respectively; chemical shifts are quoted in ppm and are relative to TMS (^{1}H, ^{13}C, ^{29}Si) and 85% H_3PO_4 (^{31}P). FTIR spectra were recorded on a Bruker Tensor 27 spectrometer. UV/Vis/NIR spectra were recorded on a Perkin Elmer Lambda 750 spectrometer. Data were collected in 1mm path length cuvettes loaded in an MBraun UniLab glovebox and were run versus the appropriate reference solvent. Static variable-temperature magnetic moment data were recorded in an applied dc field of 0.5 T on a Quantum Design MPMS XL7 superconducting quantum interference device (SQUID) magnetometer using doubly recrystallised powdered samples. Care was taken to ensure complete thermalisation of the sample before each data point was measured and samples were immobilised in an eicosane matrix to prevent sample reorientation during measurements. Diamagnetic corrections were applied for using tabulated Pascal constants and measurements were corrected for the effect of the blank sample holders (flame sealed Wilmad NMR tube and straw) and eicosane matrix. CHN microanalyses were carried out by Mr M Jennings at the University of Manchester and by Tong Liu at the University of Nottingham.

Preparation of KBi(SiMe$_3$)$_2$

To a solution of Bi(SiMe$_3$)$_3$ (18.0 g, 42.00 mmol) in THF (10 ml), KOBu^t (4.80 g, 42.0 mmol) was added at −65 °C over 20 minutes. The slightly brown solution turned orange as the solution was allowed to reach room temperature. The mixture was stirred for further 4 h, whereupon the solution further turned dark red. After removal of all volatiles under reduced pressure, the resulting solid was washed with *n*-hexane (3 × 30 ml) and was then dried under reduced pressure. Yield: 13.8 g, 81.0%. ^{1}H NMR (CD$_3$CN, 298 K): *δ* 0.62 (s, Si(C*H*$_3$)$_2$). ^{13}C{^{1}H} NMR (CD$_3$CN, 298 K): δ 12.09 (s, J_{SiC} = 36.3 Hz, *Si*(CH$_3$)$_2$).

Preparation of [Th(Tren^{DMBS})(DME)][BPh$_4$]

DME (25 ml) was added to a cold (−78 °C) mixture of [Th(Tren^{DMBS})cyclomet] (1.434 g, 2.0 mmol) and [HNEt$_3$][BPh$_4$] (0.843 g, 2.0 mmol). The colourless solution was allowed to warm to room temperature whilst stirring for 30 minutes, and stirred for a further 3 hours. Volatiles were removed *in vacuo* to give a white solid. The product was washed with pentane (3 x 5 ml) and dried under vacuum to give a fine white powder. Yield: 1.538 g (68 %). Anal. Calcd for $C_{52}H_{84}BN_4O_2Si_3Th$: C, 55.55; H, 7.53; N, 4.98%. Found: C, 55.17; H, 7.80; N, 4.48%. ^{1}H NMR (C$_6$D$_6$, 298 K): δ 0.01 (s 18H, Si(C*H*$_3$)$_2$), 0.89 (s, 27H, C(C*H*$_3$)$_3$), 2.33 (t, $^3J_{HH}$ = 4.66 Hz, 6H, C*H*$_2$CH$_2$), 2.77 (s, 4H, CH$_3$OC*H*$_2$), 2.84 (s, 6H, C*H*$_3$OCH$_2$), 3.27 (t, $^3J_{HH}$ = 4.84 Hz, 6H, CH$_2$C*H*$_2$) 7.22 (t, $^3J_{HH}$ = 7.09 Hz, 4H, *p*-Ar-*H*), 7.39 (t, $^3J_{HH}$ = 7.34 Hz, 8H, *m*-Ar-*H*), 8.06 (br, 8H, *o*-Ar-*H*). ^{13}C{^{1}H} NMR (C$_6$D$_6$, 298 K): δ −3.85 (Si(C*H*$_3$)$_2$), 20.66 (*C*(CH$_3$)$_3$), 28.20 (C(*C*H$_3$)$_3$), 47.28 (*C*H$_2$ CH$_2$), 60.95 (CH$_3$O*CH*$_2$), 62.98 (*C*H$_3$OCH$_2$), 73.58 (CH$_2$*C*H$_2$), 122.97 (*p*-Ar-*C*H,), 126.82 (*m*-Ar-*C*H), 137.50 (*o*-Ar-*C*H).^{29}Si{^{1}H} NMR (C$_6$D$_6$, 298 K): δ 3.98 (*Si*(C(CH$_3$)(CH$_3$)$_2$). FTIR (cm^{-1}): 3055 (w), 2926 (w), 2853 (w), 1580 (w), 1469 (w), 1248 (m), 1084 (m), 1023 (m), 918 (m), 801 (s), 732 (s), 702 (s), 660 (m), 611 (m), 447.21 (m).

Preparation of [U(Tren^{DMBS}){P(SiMe$_3$)$_2$}] (3UP)

THF (30 ml) was added to a cold (−78 °C) mixture of [U(Tren^{DMBS})(THF)][BPh$_4$] (3.35 g, 3 mmol) and KP(SiMe$_3$)$_2$ (0.65 g, 3 mmol). The resulting red solution was allowed to warm to room

temperature and stirred for 30 min. Solvents were removed *in vacuo* and the resulting solid was extracted with pentane (2 x 20 ml) and filtered. Removal of solvents *in vacuo* afforded a red solid. Yield: 2.16 g, 80%. Red crystals suitable for single crystal XRD were obtained from a concentrated pentane solution stored at (−30 °C) for 16 hours. Anal. Calcd. for $C_{30}H_{75}N_4PSi_5U \cdot 0.36C_5H_{12}$: C 40.26; H 8.49; N 6.14%. Found: C 40.26; H 8.44; N 5.78%. ^{1}H NMR (C_6D_6, 295 K): δ −36.74 (s, 6H, CH_2CH$_2$), −13.78 (s, 18H, P{Si(CH_3)$_2$}), 7.92 (s, 6H, CH$_2$CH_2), 9.02 (s, 18H, Si(CH_3)$_2$), 13.04 (s, 27H, C(CH_3)$_3$). ^{31}P NMR (C_6D_6, 298 K): δ 2055.21 (s, *P*{Si(CH$_3$)$_2$}). ^{29}Si{^{1}H} NMR (C_6D_6, 298 K): δ no resonances observed. μ_{eff} (Evans method, C_6D_6, 298 K): 2.76 μ_B. FTIR (cm^{-1}): 2950 (s), 2926 (s), 2884 (s), 2851 (s), 1463 (s), 1239 (s), 1061 (s), 930 (s), 822 (w), 799 (s), 770 (w), 656 (w), 623 (w), 474 (w), 433 (w).

Preparation of [U(Tren^{DMBS}){As(SiMe$_3$)$_2$}] (3UAs)

THF (30 ml) was added to a cold (−78 °C) mixture of [U(Tren^{DMBS})(THF)][BPh$_4$] (3.35 g, 3 mmol) and KAs(SiMe$_3$)$_2$ (0.78 g, 3 mmol). The resulting burgundy solution was allowed to warm to room temperature and stirred for 30 min. Solvents were removed *in vacuo* and the resulting solid was extracted with pentane (2 x 20 ml) and filtered. Removal of solvents *in vacuo* afforded a burgundy solid. Yield: 1.89 g, 67%. Red crystals suitable for single crystal XRD were obtained from a concentrated pentane solution stored at (−30 °C) for 16 hours. Anal. Calcd. for $C_{30}H_{75}N_4AsSi_5U$: C, 38.12; H, 8.00; N, 5.93%. Found: C 38.06; H 7.97; N 5.58%. ^{1}H NMR (C_6D_6, 295 K): δ −36.06 (s, 6H, CH_2CH$_2$), −12.17 (s, 18H, As{Si(CH_3)$_2$}), 3.42 (b, 6H, CH$_2$CH_2), 10.32 (s, 18H, Si(CH_3)$_2$), 12.63 (s, 27H, C(CH_3)$_3$). ^{29}Si{^{1}H} NMR (C_6D_6, 298 K): δ no resonances observed. μ_{eff} (Evans method, C_6D_6, 298 K): 3.19 μ_B. FTIR (cm^{-1}): 2949 (s), 2925 (s), 2884 (s), 2851 (s), 1463 (s) 1238 (s), 1071 (s), 1060 (s), 930 (s), 906 (w) 890 (s), 821 (s) 798 (s), 770 (w), 771 (w). 675 (s), 657 (w), 618 (w), 588 (w), 565 (s), 526 (w), 446 (w).

Preparation of [U(Tren^{DMBS}){Sb(SiMe$_3$)$_2$}] (3USb)

THF (30 ml) was added to a cold (−78 °C) mixture of [U(Tren^{DMBS})(THF)][BPh$_4$] (3.35 g, 3 mmol) and KSb(SiMe$_3$)$_2$ (0.92 g, 3 mmol). The resulting dark green solution was allowed to warm to room

temperature and stirred for 30 min. Solvents were removed *in vacuo* and the resulting solid was extracted with pentane (2 x 20 ml) and filtered. Removal of solvents *in vacuo* afforded a dark green solid. Yield: 2.40 g, 81%. Dark green crystals suitable for single crystal XRD were obtained from a concentrated hexane solution stored at (−30 °C) for 15 hours. Anal. Calcd. for $C_{30}H_{75}N_4SbSi_5U$: C 36.32; H 7.62; N 5.65%. Found: C 36.72; H 7.58; N 5.42%. ^{1}H NMR (C_6D_6, 295 K): δ −36.30 (s, 6H, CH_2CH$_2$), −9.95 (s, 18H, Sb{Si(CH_3)$_2$}), −3.59 (s, 6H, CH$_2$CH_2), 12.41 (s, 27H, C(CH_3)$_3$), 13.00 (s, 18H, Si(CH_3)$_2$). ^{29}Si{^{1}H} NMR (C_6D_6, 298 K): δ no resonances observed. μ_{eff} (Evan's Method, C_6D_6, 298 K): 2.86 μ_B. FTIR (cm^{-1}): 2929 (w), 2926 (w), 2885 (s), 2852 (s), 1463 (s), 1389 (w), 1359 (w), 1347 (w), 1285 (w), 1248 (s), 1235 (s), 1138 (w), 1083 (w), 1071 (s), 940 (w), 923 (s), 902 (w), 798 (s), 770 (w), 697 (w), 677 (s), 615 (w), 590 (w), 565 (w), 529 (w), 448 (w), 409(w).

Preparation of [U(Tren^{DMBS}){Bi(SiMe$_3$)$_2$}] (3UBi)

THF (30 ml) was added to a cold (−78 °C) mixture of [U(Tren^{DMBS})(THF)][BPh$_4$] (3.35 g, 3 mmol) and KBi(SiMe$_3$)$_2$ (1.18 g, 3 mmol). The resulting green solution was allowed to warm to room temperature and stirred for 30 min. Solvents were removed *in vacuo* and the resulting solid was extracted with pentane (2 x 20 ml) and filtered. Removal of solvents *in vacuo* afforded a green crystalline solid. Yield: 1.95 g, 60%. Green crystals were obtained from a concentrated hexane solution stored at (−30 °C) for 15 hours. Anal. Calcd. for $C_{30}H_{75}N_4BiSi_5U$: C 33.38; H 7.00; N 5.19%. Found: C 32.94; H 6.90; N 4.99%. ^{1}H NMR (C_6D_6, 295 K): δ −36.74 (s, 6H, CH_2CH$_2$), −9.37 (s, 18H, Sb{Si(CH_3)$_2$}), −2.26 (s, 6H, CH$_2$CH_2), 12.63 (s, 27H, C(CH_3)$_3$), 13.27 (s, 18H, Si(CH_3)$_2$). ^{29}Si{^{1}H} NMR (C_6D_6, 298 K): δ no resonances observed. μ_{eff} (Evan's Method, C_6D_6, 298 K): 2.80 μ_B. FTIR (cm^{-1}): 2951 (s), 2926 (s), 2882 (s), 2851 (s), 1463 (s), 1388 (s), 1359 (s), 1347 (s), 1249 (s), 1071 (s), 1056 (s), 1021 (s), 924 (s), 900 (s) 796 (s), 770 (s), 705 (s), 677 (w), 659 (s), 616 (s), 561 (w), 529 (w), 450 (s).

Preparation of [U(Tren^{TIPS}){P(SiMe$_3$)$_2$}] (4UP)

THF (30 ml) was added to a cold (−78 °C) mixture of [U(Tren^{TIPS})(THF)][BPh$_4$] (3.72 g, 3.0 mmol) and KP(SiMe$_3$)$_2$ (0.65 g, 3.0 mmol). The resulting red solution was allowed to warm to room

temperature and stirred for 30 min. Solvents were removed *in vacuo* and the resulting solid was extracted into toluene (2 × 20 ml) and filtered. Removal of solvent *in vacuo* afforded a red solid. Yield: 2.53 g, 81%. Red crystals suitable for single crystal XRD were obtained from a concentrated hexane solution stored at −30 °C for 16 hours. Anal. Calcd. for $C_{39}H_{93}N_4PSi_5U{\cdot}0.2(C_7H_8)$: C 46.39; H 9.12; N 5.36%. Found: C 46.48; H 9.19; N 5.26%. ^{1}H NMR (C_6D_6, 298 K): δ −41.29 (s, 6H, CH_2CH_2), −17.25 (s, 18H, P{Si(CH_3)$_2$}), 1.44 (s, 6H, CH_2CH_2), 13.41 (9H, s, $CH(CH_3)_3$), 15.50 (s, 54H, $CH(CH_3)_2$). ^{31}P NMR (C_6D_6, 298 K): δ no resonances observed. ^{29}Si{^{1}H} NMR (C_6D_6, 298 K): δ −97.09 (*Si*Me$_3$)$_2$), 42.96 (*Si*(CH(CH$_3$)$_2$)$_3$). μ_{eff} (Evans method, C_6D_6, 298 K): 3.22 μ_B. FTIR (cm^{-1}): 1959 (w), 1463 (s), 1302 (w), 1273 (w), 1239 (s), 1140 (s), 1035 (s), 1010 (s), 930 (s), 902 (w), 881 (s), 828 (s), 806 (w), 726 (s), 671 (s), 656 (s), 627 (s), 568 (s), 518 (s), 465 (s), 431 (s).

Preparation of [U(Tren^{TIPS}){As(SiMe$_3$)$_2$}] (4UAs)

THF (30 ml) was added to a cold (−78 °C) mixture of [U(Tren^{TIPS})(THF)][BPh$_4$] (3.72 g, 3 mmol) and KAs(SiMe$_3$)$_2$ (0.78 g, 3 mmol). The resulting burgundy solution was allowed to warm to room temperature and stirred for 30 min. Solvents were removed *in vacuo* and the resulting solid was extracted with pentane (2 × 20 ml). The green solution was filtered, solvents were removed *in vacuo* and the resulting solid was recrystallized from HMDSO to obtain an analytically pure sample. Yield: 1.86 g, 58%. Dark red crystals suitable for single crystal XRD were obtained from a concentrated benzene solution stored at 5 °C for 6 hours. Anal. Calc'd. for $C_{39}H_{93}AsN_4Si_5U$: C 43.71; H 8.75; N 5.23%. Found: C 43.83; H 8.63; N 5.28%. ^{1}H NMR (C_6D_6, 298 K): δ −39.14 (s, 6H, CH_2CH_2), –14.23 (s, 18H, As{Si(CH_3)$_2$}), 1.41 (s, 6H, CH_2CH_2), 12.45 (9H, s, $CH(CH_3)_2$), 14.33 (s, 54H, $CH(CH_3)_2$). ^{29}Si{^{1}H} NMR (C_6D_6, 298 K): δ −61.03 (*Si*Me$_3$)$_2$), 38.68 (*Si*(CH(CH$_3$)$_2$)$_3$). μ_{eff} (Evans method, C_6D_6, 298 K): 3.27 μ_B. FTIR (cm^{-1}): 2939 (s), 2889 (w), 2862 (s), 1461 (s), 1381 (w), 1362 (w), 1340 (w), 1274 (s), 1236 (s), 1139 (s), 1065 (w), 1051 (s), 1035 (s), 1010 (w), 931 (s), 907 (w), 879 (s), 848 (w), 820 (s), 806 (s), 717 (s), 671 (s), 622 (s), 566 (s), 556 (s), 516 (s).

Preparation of [U(Tren^{TIPS}){Sb(SiMe$_3$)$_2$}] (4USb)

THF (30 ml) was added to a cold (−78 °C) mixture of [U(Tren^{TIPS})(THF)][BPh$_4$] (3.72 g, 3 mmol) and KSb(SiMe$_3$)$_2$ (0.92 g, 3 mmol). The resulting dark green solution was allowed to warm to room temperature and stirred for 30 min. Solvents were removed *in vacuo* and the resulting solid was extracted with pentane (2 × 20 ml). The green solution was filtered and solvents were removed *in vacuo* yielding a green solid. Yield: 1.86 g, 62%. Dark green crystals suitable for single crystal XRD were obtained from a concentrated hexane solution stored at −30 °C for 16 hours. Anal. Calc'd. for $C_{39}H_{93}N_4SbSi_5U\cdot 0.25C_6H_{14}$: C 42.67; H 8.53; N 4.91%. Found: C 42.52; H 8.37; N 4.74%. ^{1}H NMR (C_6D_6, 298 K): δ −39.00 (s, 6H, C*H*$_2$CH$_2$), –10.20 (s, 18H, Sb{Si(C*H*$_3$)$_2$}), 0.95 (s, 6H, CH$_2$C*H*$_2$), 11.26 (9H, s, C*H*(CH$_3$)$_2$), 13.47 (s, 54H, CH(C*H*$_3$)$_2$). ^{29}Si{^{1}H} NMR (C_6D_6, 298 K): δ −30.78 (*Si*Me$_3$), 38.17 (*Si*(CH(CH$_3$)$_2$)$_3$). μ_{eff} (Evans method, C_6D_6, 298 K): 2.89 μ_B. FTIR (cm^{-1}): 2941 (s), 2888 (w), 2861 (s), 1461 (s), 1382 (w), 1364 (w), 1336 (w), 1272 (s), 1233 (s), 1140 (w), 1032 (w), 1009 (s), 928 (s), 880 (s), 823 (s), 803 (s), 712 (s), 674 (s), 621 (s), 573 (s), 517 (s), 446 (s).

Preparation of [Th(Tren^{DMBS}){P(SiMe$_3$)$_2$}] (3ThP)

DME (25 ml) was added to a cold (−78 °C) mixture of [Th(Tren^{DMBS})(DME)][BPh$_4$] (0.769 g, 0.68 mmol) and KP(SiMe$_3$)$_2$ (0.147 g, 0.68 mmol). The pale yellow solution was allowed to warm to room temperature whilst stirring for 30 minutes. Volatiles were removed *in vacuo* and the product was extracted into pentane. Crystalline material was obtained from a toluene (3 ml) solution stored at −30 °C. Yield: 0.532g, 87%. Anal. Calcd for $C_{30}H_{75}N_4PSi_5Th$: C, 40.24; H, 8.44; N, 6.26%. Found: C, 39.05; H, 8.01; N, 5.85%.^{1}H NMR (C_6D_6, 298 K): δ 0.49 (s, 18H, Si(C*H*$_3$)$_2$), 0.62 (d, $^3J_{PH}$ = 4.16 Hz, 18H, P{Si(C*H*$_3$)$_2$}), 1.03 (s, 27H, C(C*H*$_3$)$_3$), 2.45 (t, $^3J_{HH}$ = 5.14 Hz, 6H, C*H*$_2$CH$_2$), 3.36 (t, $^3J_{HH}$ = 5.01 Hz, 6H, CH$_2$C*H*$_2$). ^{31}P NMR (C_6D_6, 298 K): δ −100.09 (s, *P*(SiMe$_3$)$_2$). ^{13}C{^{1}H} NMR (C_6D_6, 298 K): δ −3.69 (Si(C*H*$_3$)$_2$), 7.02 (d, $^2J_{CP}$ = 9.54 Hz, (PSi(*C*H$_3$)$_2$), 21.25 (*C*(CH$_3$)$_3$), 28.52 (C(*C*H$_3$)$_3$), 47.81 (*C*H$_2$ CH$_2$), 63.25 (CH$_2$*C*H$_2$). ^{29}Si{^{1}H} NMR (C_6D_6, 298 K): δ no resonances observed. FTIR (cm^{-1}): 2949 (m), 2889 (m), 2849 (m), 1469 (w), 1387 (m), 1245 (m), 1074 (m), 924 (s), 895 (s), 797 (s), 783 (s) 769 (s), 709 (s), 657 (m), 560 (m), 454 (m).

Preparation of [Th(Tren^{DMBS}){As(SiMe$_3$)$_2$}] (3ThAs)

DME (25 ml) was added to a cold (−78 °C) mixture of [Th(Tren^{DMBS})(DME)][BPh_4] (1.13 g, 1.0 mmol) and KAs($SiMe_3$)$_2$ (0.26 g, 1.0 mmol). The pale yellow solution was allowed to warm to room temperature whilst stirring for 4 minutes. Volatiles were removed *in vacuo* and the product was extracted into pentane. Crystalline material was obtained from a pentane (3 ml) solution stored at room temperature. Yield: 0.316 g, 34%. Anal. Calcd for $C_{30}H_{75}N_4AsSi_5Th$: C, 38.36; H, 8.05; N, 5.96%. Found: C, 38.06; H, 8.17; N, 5.82%. ^{1}H NMR (C_6D_6, 298 K): δ 0.50 (s, 18H, Si(CH_3)$_2$), 0.70 (s, 18H, As{Si(CH_3)$_2$}), 1.03 (s, 27H, C(CH_3)$_3$), 2.43 (t, $^3J_{HH}$ = 5.26 Hz, 6H, CH_2CH$_2$), 3.34 (t, $^3J_{HH}$ = 5.31 Hz, 6H, CH$_2$CH_2). ^{13}C{^{1}H} NMR (C_6D_6, 298 K): δ −3.70 (Si(CH_3)$_2$), 7.78 (AsSi(CH$_3$)$_2$), 21.24 (C(CH$_3$)$_3$), 28.50 (C(CH$_3$)$_3$), 47.73 (CH$_2$CH$_2$), 63.76 (CH$_2$$CH_2$). ^{29}Si{^{1}H} NMR ($C_6D_6$, 298 K): δ −35.55 ($Si$(CH(CH$_3$)$_2$)$_3$), 8.78 (($SiMe_3$)$_2$). FTIR (cm^{-1}): 2950 (m), 2925 (m), 2887 (m), 2851 (m), 1246 (m), 1078 (m), 925 (s), 819 (s), 798 (s),) 769 (s), 739 (m), 655(m), 617 (m), 545 (w).

Preparation of [Th(Tren^{TIPS}){P(SiMe$_3$)$_2$}] (4ThP)

DME (25 ml) was added to a cold (−78 °C) mixture of [Th(Tren^{TIPS})(DME)][BPh_4] (0.493 g, 0.4 mmol) and KP($SiMe_3$)$_2$ (0.087 g, 0.4 mmol). The pale yellow solution was allowed to warm to room temperature whilst stirring for 30 minutes. Volatiles were removed *in vacuo* and the product was extracted into toluene. Crystalline material was obtained from a toluene (3 ml) solution stored at −30 °C. Yield: 0.122 g (30%). Anal. Calcd for $C_{39}H_{93}N_4PSi_5Th{\bullet}0.15C_7H_8$: C, 46.46; H, 9.17; N, 5.41%. Found: C, 46.49; H, 9.02; N, 5.44%. ^{1}H NMR (C_6D_6, 298 K): δ 0.59 (d, $^3J_{PH}$ = 4.03 Hz, 18H, Si(CH_3)$_2$), 1.30 (d, $^3J_{HH}$ = 7.09 Hz, 54H, CH(CH_3)$_3$), 1.44 (septet, $^3J_{HH}$ = 7.70, 9H, CH(CH$_3$)$_3$), 2.50 (t, $^3J_{HH}$ = 4.28 Hz, 6H, CH_2CH$_2$), 3.60 (t, $^3J_{HH}$ = 4.28 Hz, 6H, CH$_2$CH_2). ^{13}C{^{1}H} NMR (C_6D_6, 298 K): δ 8.09 (d, $^2J_{PC}$ = 9.53 Hz, Si(CH_3)$_2$), 14.93 (CH(CH$_3$)$_2$), 20.81 (CH(CH$_3$)$_2$), 46.14 (CH$_2$), 64.95 (CH$_2$). ^{31}P NMR (C_6D_6, 298 K): δ −66.45 (s, P(SiMe$_3$)$_2$). ^{29}Si{^{1}H} NMR (C_6D_6, 298 K): δ 5.12 (Si(CH(CH$_3$)$_2$)$_3$), 8.49 ((SiMe$_3$)$_2$). FTIR (cm^{-1}): 2940 (m), 2888 (m), 2862 (m), 1460 (m), 1381 (w), 1237 (m), 1137 (w), 1039 (m), 987 (m), 929 (s), 880 (s), 825 (s), 728 (s) 671 (s), 624 (s), 562 (m), 516 (m), 432 (m).

Preparation of [Th(Tren^{TIPS}){As(SiMe$_3$)$_2$}] (4ThAs)

DME (25 ml) was added to a cold (−78 °C) mixture of [Th(Tren^{TIPS})(DME)][BPh_4] (1.23 g, 1.0 mmol) and KAs($SiMe_3$)$_2$ (0.260 g, 1.0 mmol). The pale yellow solution was allowed to warm to room temperature whilst stirring for 30 minutes. Volatiles were removed *in vacuo* and the product was extracted into pentane. Crystalline material was obtained from a pentane (3 ml) solution stored at −30 °C. Yield: 0.204 g (19%). Anal. Calcd for $C_{39}H_{93}AsN_4Si_5Th$: C, 43.96; H, 8.80; N, 5.26%. Found: C, 44.37; H, 8.58; N, 5.52%. ^{1}H NMR (C_6D_6, 298 K): δ 0.68 (s, 18H, Si(C*H*$_3$)$_2$), 1.30 (d, $^3J_{HH}$ = 7.21 Hz, 54H, CH(C*H*$_3$)$_3$), 1.45 (septet, $^3J_{HH}$ = 7.04 Hz, 9H, C*H*(CH$_3$)$_3$), 2.51 (t, br, 6H, C*H$_2$*CH$_2$), 3.61 (t, br, 6H, CH$_2$C*H$_2$*). ^{13}C{^{1}H} NMR (C_6D_6, 298 K): δ 8.80 (Si(C*H*$_3$)$_2$), 14.81 (*C*H(CH$_3$)$_2$), 20.79 (CH(*C*H$_3$)$_2$), 46.28 (*C*H$_2$), 65.08 (*C*H$_2$). ^{29}Si{^{1}H} NMR (C_6D_6, 298 K): δ 5.20 (*Si*(CH(CH$_3$)$_2$)$_3$), 11.46 ((*Si*Me$_3$)$_2$). FTIR (cm^{-1}): 2939 (m), 2889 (m), 2861 (m), 1462 (w), 1382 (m), 1237 (m), 1041 (m), 1010 (m) 929 (s), 880 (s), 810 (s), 730 (s) 672 (s), 625 (s), 564 (m), 516 (m), 446 (m).

Preparation of [Th(Tren^{TIPS}){Sb(SiMe$_3$)$_2$}] (4ThSb)

DME (25 ml) was added to a cold (−78 °C) mixture of [Th(Tren^{TIPS})(DME)][BPh_4] (0.911 g, 0.73 mmol) and KSb($SiMe_3$)$_2$ (0.223 g, 0.73 mmol) to give a brown solution. Volatiles were immediately removed *in vacuo* and the product was extracted into pentane (10 ml), giving a dark orange solution. Crystalline material was obtained from a pentane (3 ml) solution stored at −30 °C. Yield: 0.112 g (14%). Anal. Calcd for $C_{39}H_{93}N_4SbSi_5Th$: C, 42.11; H, 8.43; N, 5.04%. Found: C, 41.87; H, 8.60; N, 5.15%.^{1}H NMR (C_6D_6, 298 K): δ 0.82 (s, 18H, Si(C*H*$_3$)$_2$), 1.30 (d, $^3J_{HH}$ = 7.34 Hz, 54H, CH(C*H*$_3$)$_3$), 1.45 (septet, $^3J_{HH}$ = 7.43 Hz, 9H, C*H*(CH$_3$)$_3$), 2.51 (t, $^3J_{HH}$ = 4.27 Hz, 6H, C*H$_2$*CH$_2$), 3.59 (t, $^3J_{HH}$ = 4.48 Hz, 6H, CH$_2$C*H$_2$*). ^{13}C{^{1}H} NMR (C_6D_6, 298 K): δ 10.10 (Si(C*H*$_3$)$_2$), 14.51 (*C*H(CH$_3$)$_2$), 20.76 (CH(*C*H$_3$)$_2$), 46.52 (*C*H$_2$), 64.66 (*C*H$_2$). ^{29}Si{^{1}H} NMR (C_6D_6, 298 K): δ 5.38 (*Si*(CH(CH$_3$)$_2$)$_3$). (Sb(*Si*Me$_3$)$_2$) resonance not observed. FTIR (cm^{-1}): 2940 (m), 2887 (m), 2861 (m), 1461 (w), 1381 (w), 1233 (m), 1039 (m), 1011 (m) 927 (s), 880 (s), 808 (s), 727 (s) 673 (s), 630 (s), 570 (m), 516 (m), 446 (m).

Magnetism

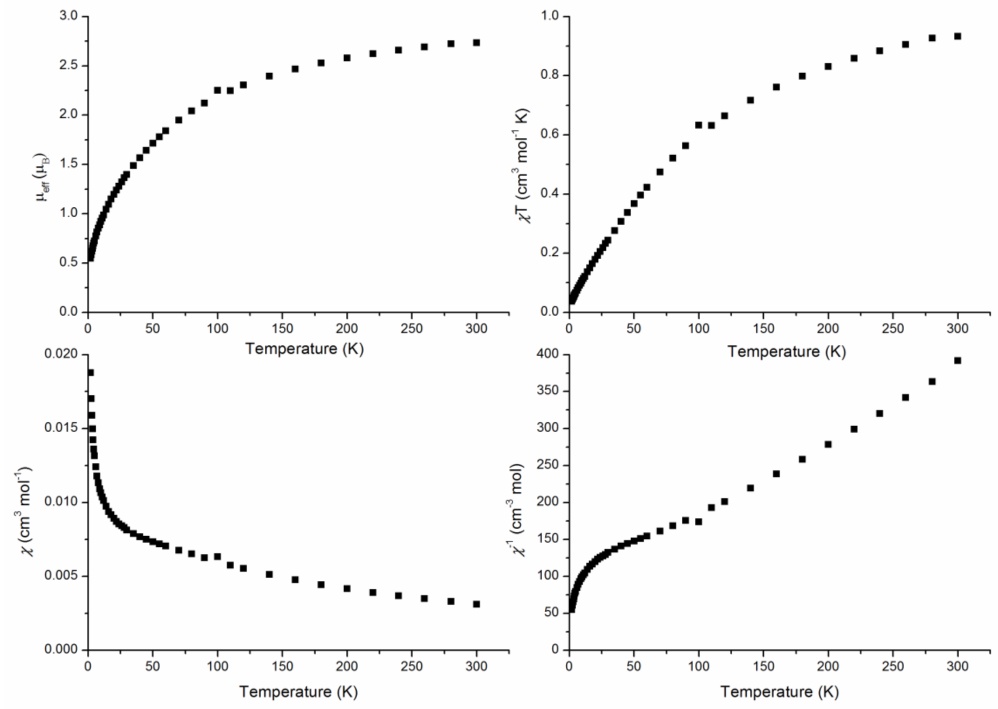

Figure S1. Variable Temperature SQUID Data for μeff vs T (top left, μ_{eff} = 2.73 (298 K) and 0.55 (2 K μ_B), χT vs T (top right), χ vs T (bottom left), and 1/χ vs T (bottom right) for **3UP**.

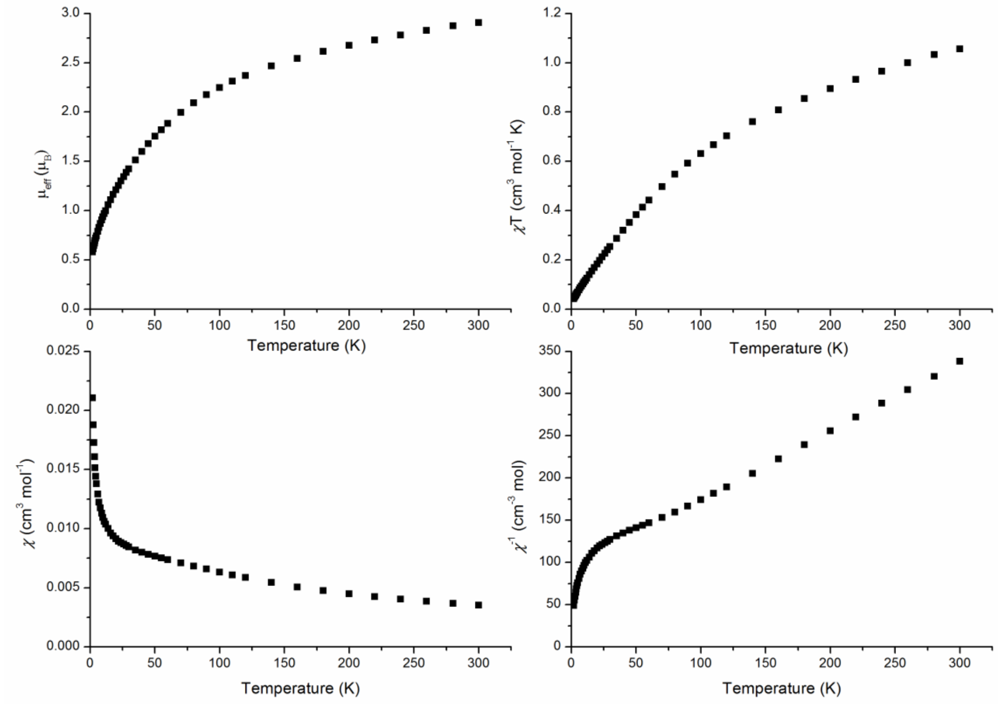

Figure S2. Variable Temperature SQUID Data for μeff vs T (top left, μ_{eff} = 2.91 (298 K) and 0.58 (2 K μ_B), χT vs T (top right), χ vs T (bottom left), and 1/χ vs T (bottom right) for **3UAs**.

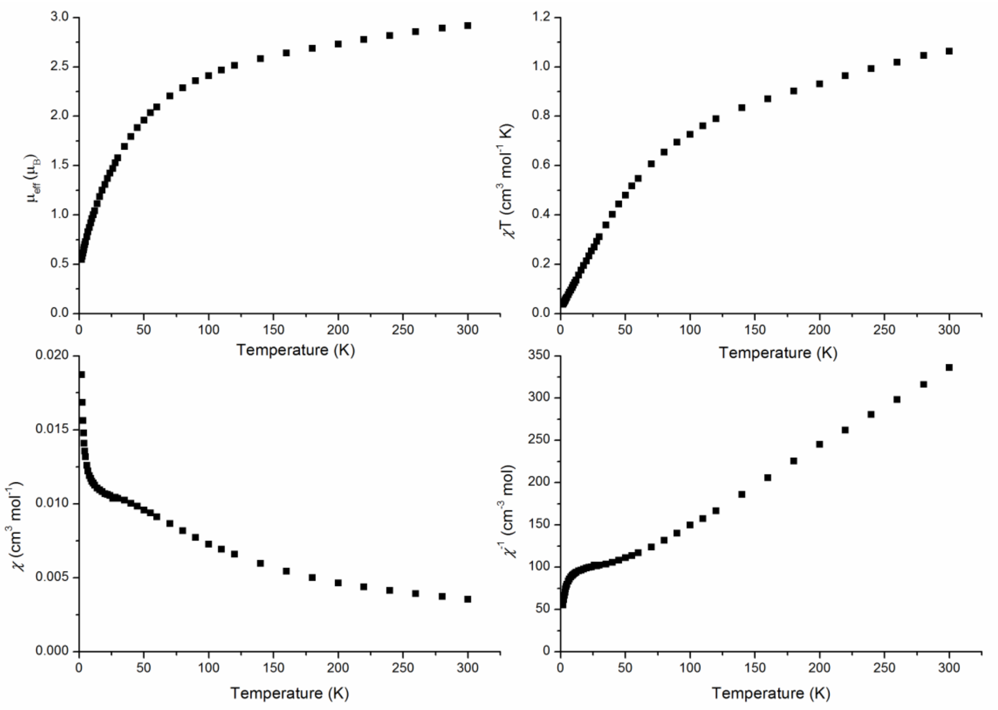

Figure S3. Variable Temperature SQUID Data for μeff vs T (top left, μ_{eff} = 2.92 (298 K) and 0.55 (2 K μ_B), χT vs T (top right), χ vs T (bottom left), and 1/χ vs T (bottom right) for **3USb**.

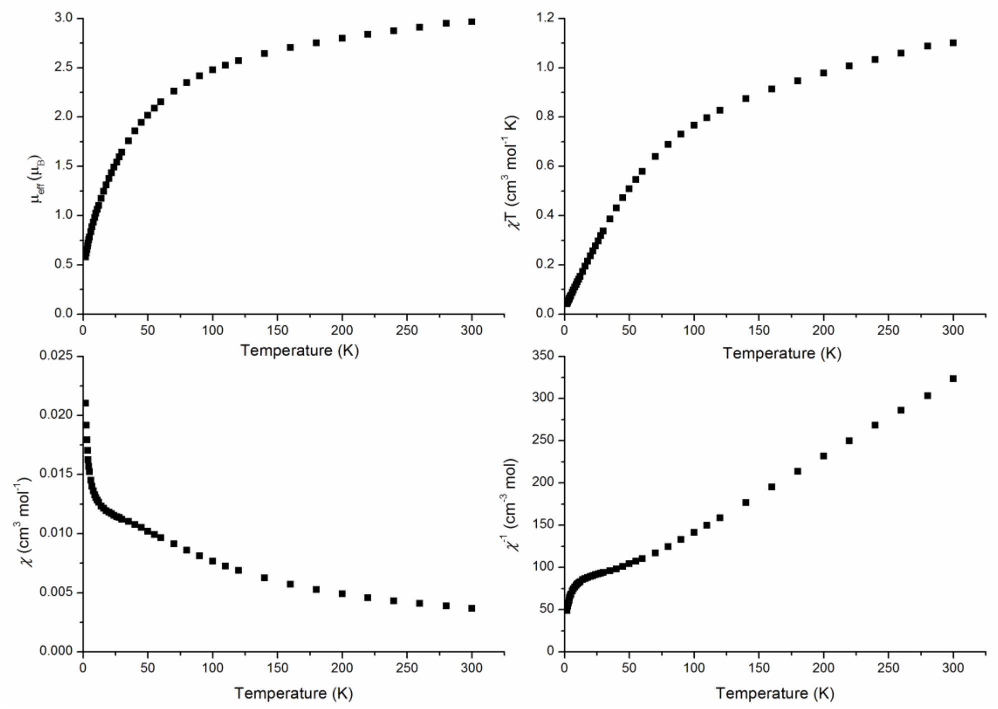

Figure S4. Variable Temperature SQUID Data for μeff vs T (top left, μ_{eff} = 2.97 (298 K) and 0.58 (2 K μ_B), χT vs T (top right), χ vs T (bottom left), and 1/χ vs T (bottom right) for **3UBi**.

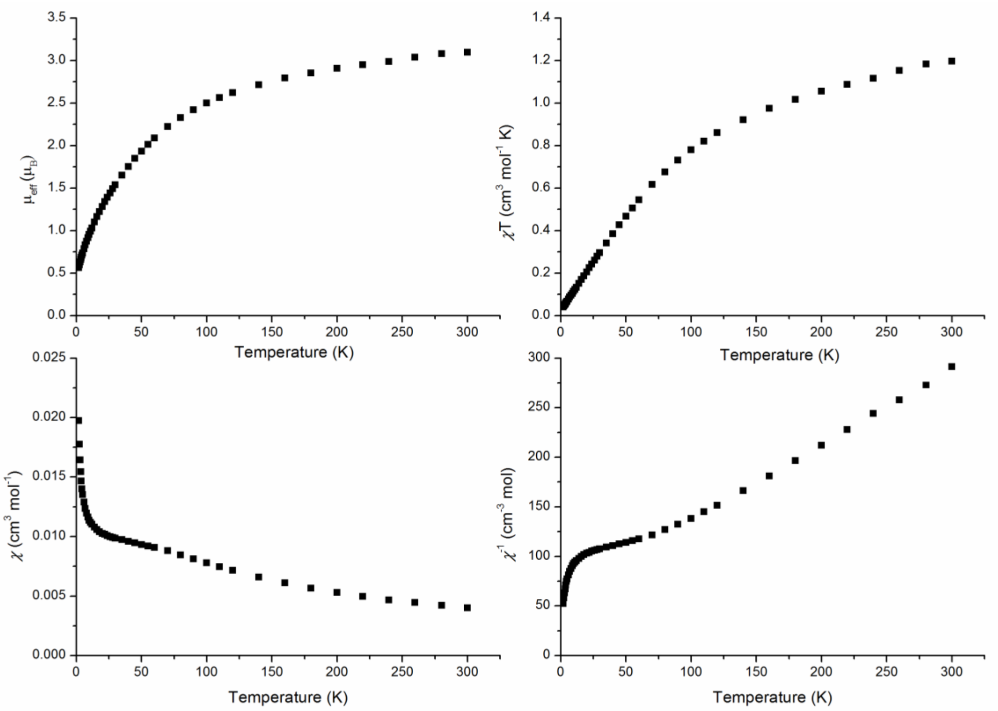

Figure S5. Variable Temperature SQUID Data for μeff vs T (top left, μ_{eff} = 3.09 (298 K) and 0.56 (2 K μ_B), χT vs T (top right), χ vs T (bottom left), and 1/χ vs T (bottom right) for **4UP**.

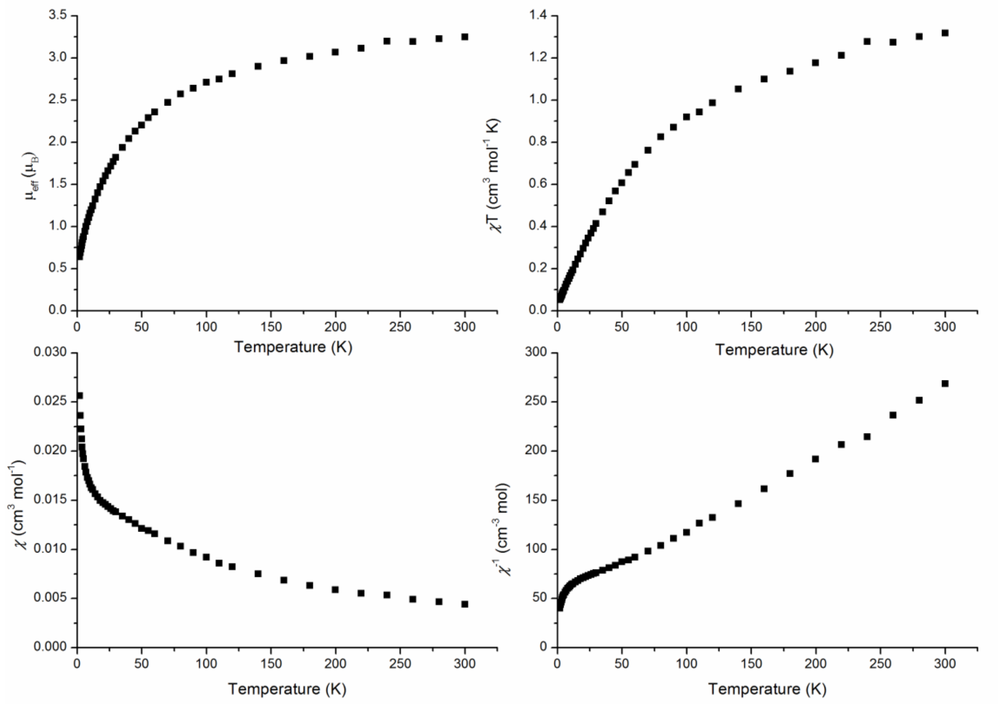

Figure S6. Variable Temperature SQUID Data for μeff vs T (top left, μ_{eff} = 3.25 (298 K) and 0.64 (2 K μ_B), χT vs T (top right), χ vs T (bottom left), and 1/χ vs T (bottom right) for **4UAs**.

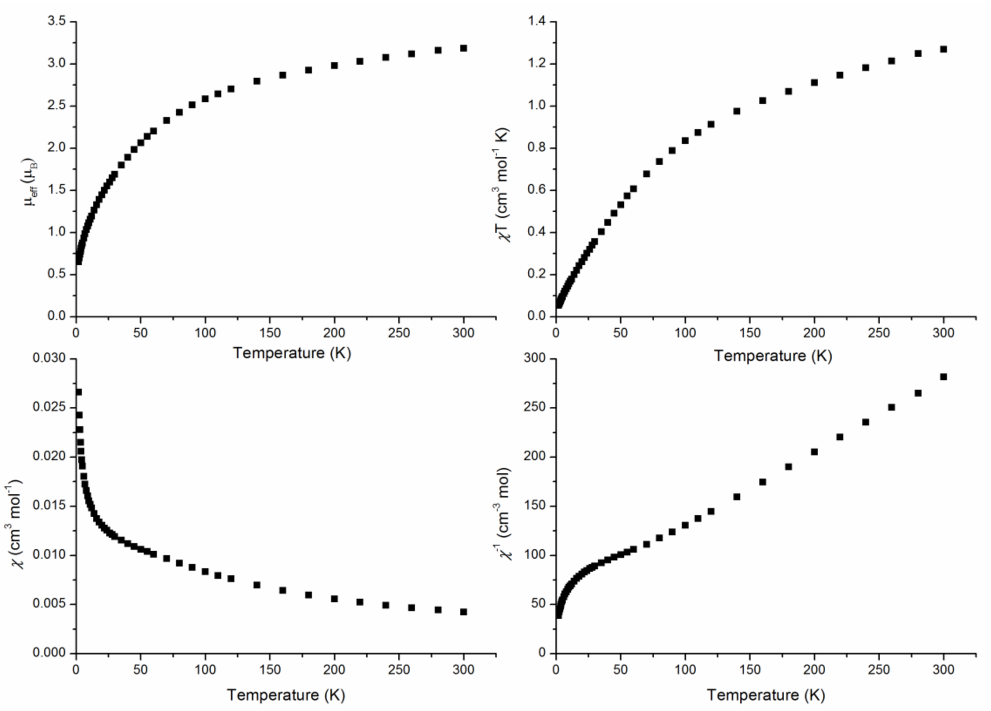

Figure S7. Variable Temperature SQUID Data for μeff vs T (top left, μ_{eff} = 2.86 (298 K) and 0.65 (2 K μ_B), χT vs T (top right), χ vs T (bottom left), and 1/χ vs T (bottom right) for **4USb**.

UV/Vis/NIR Electronic Absorption Spectrum

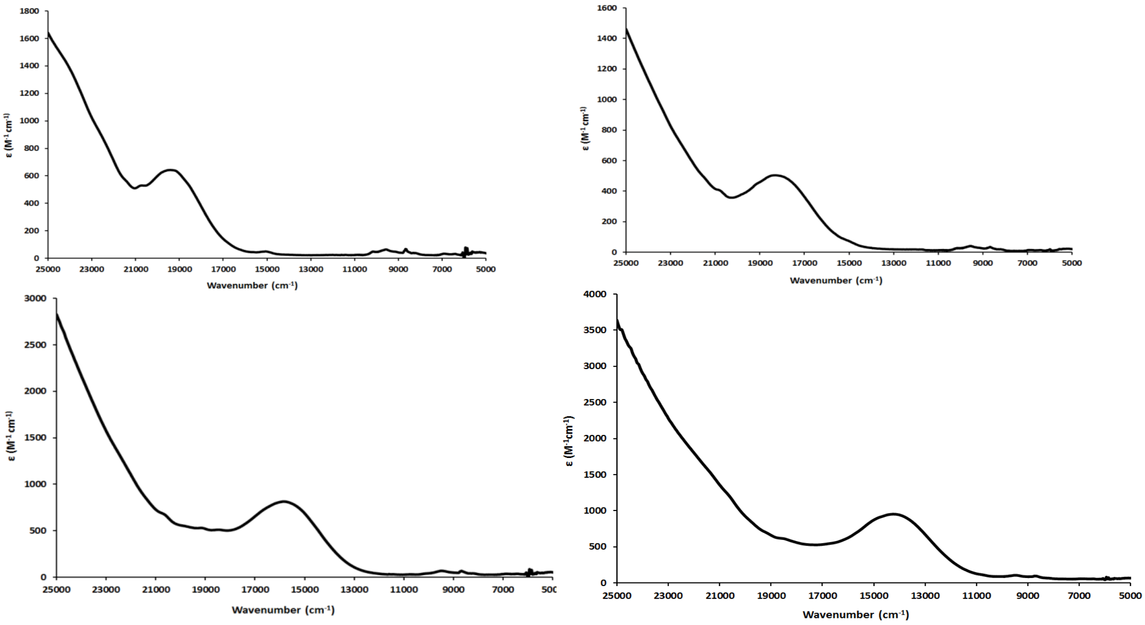

Figure S8. UV/VIS/NIR electronic absorption spectra of **3UP** (top left), **3UAs** (top right), **3USb** (bottom left), and **3UBi** (bottom right), 10mM in Toluene in 1mm cell.

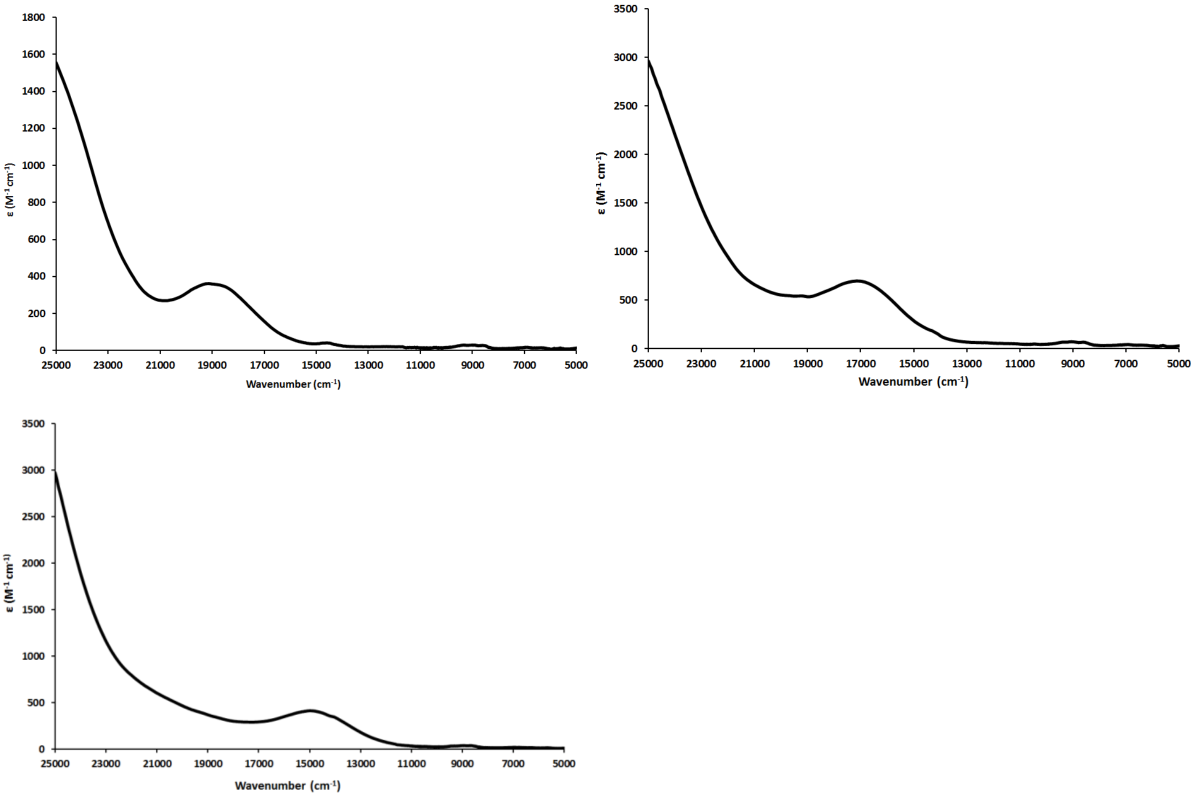

Figure S9. UV/VIS/NIR electronic absorption spectra of **4UP** (top left), **4UAs** (top right), and **4USb** (bottom left), 10mM in Toluene in 1mm cell.

X-ray Crystallography (CCDC 1585332-1585346**)**

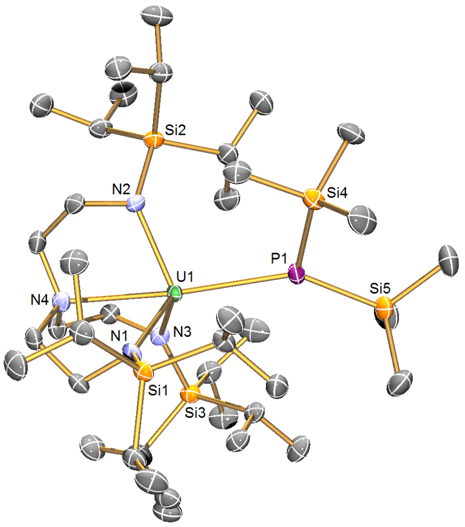

Figure S10. Solid state structure of **4UP** at 150 K and displacement ellipsoids set to 40% and hydrogen atoms, minor disorder components, and any lattice solvent omitted for clarity.

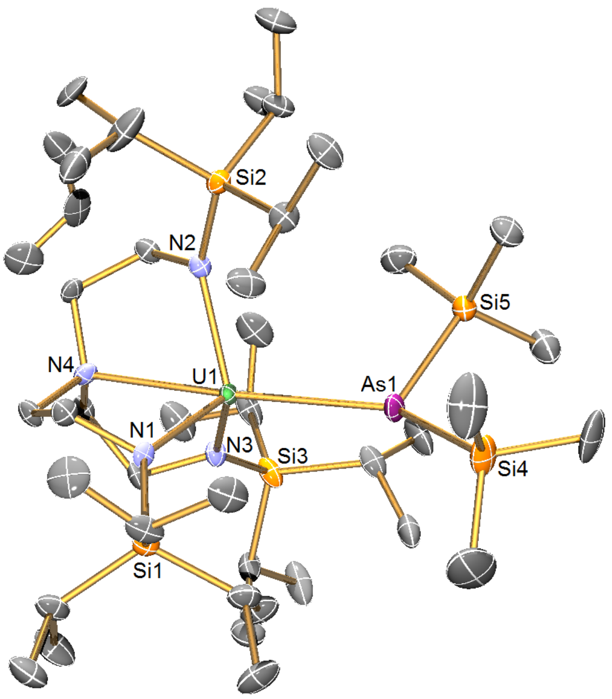

Figure S11. Solid state structure of **4UAs** at 150 K and displacement ellipsoids set to 40% and hydrogen atoms, minor disorder components, and any lattice solvent omitted for clarity.

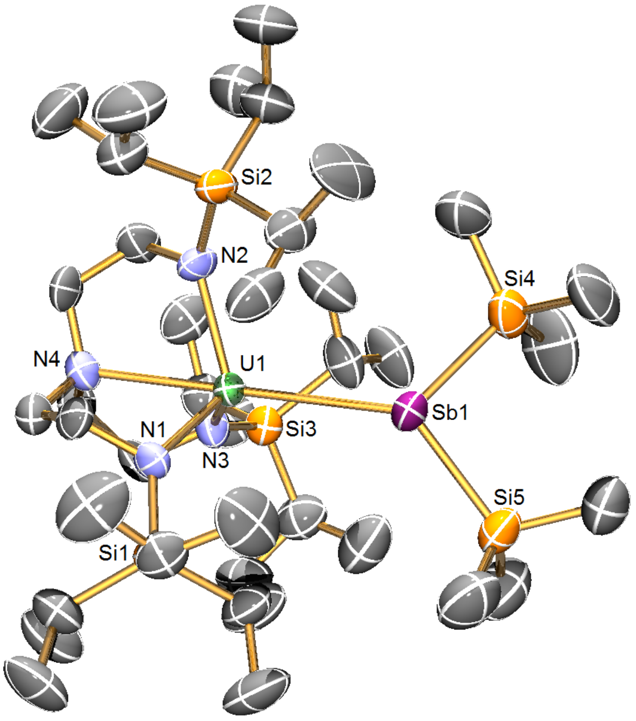

Figure S12. Solid state structure of **4USb** at 120 K and displacement ellipsoids set to 40% and hydrogen atoms, minor disorder components, and any lattice solvent omitted for clarity.

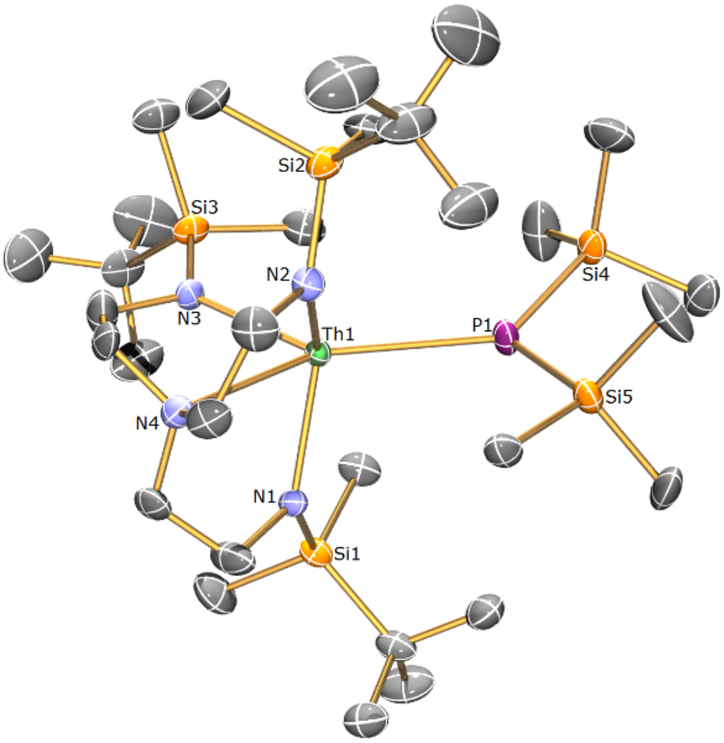

Figure S13. Solid state structure of **3ThP** at 150 K and displacement ellipsoids set to 40% and hydrogen atoms, minor disorder components, and any lattice solvent omitted for clarity.

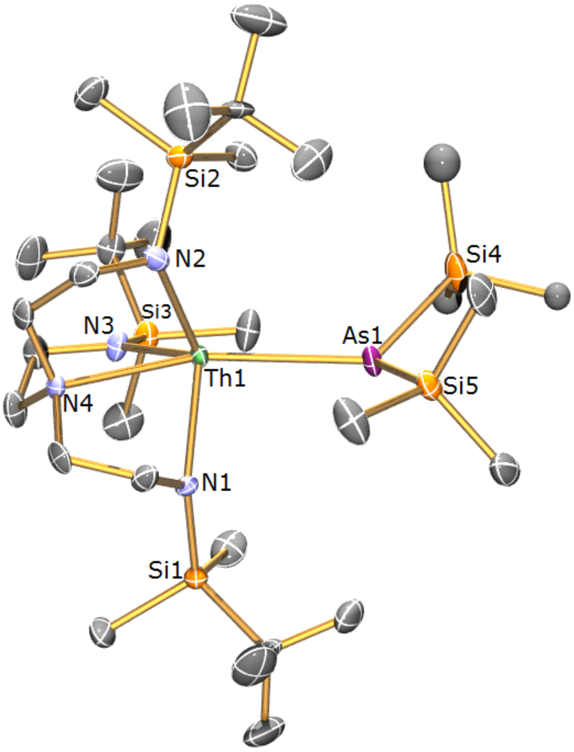

Figure S14. Solid state structure of **3ThAs** at 150 K and displacement ellipsoids set to 40% and hydrogen atoms, minor disorder components, and any lattice solvent omitted for clarity.

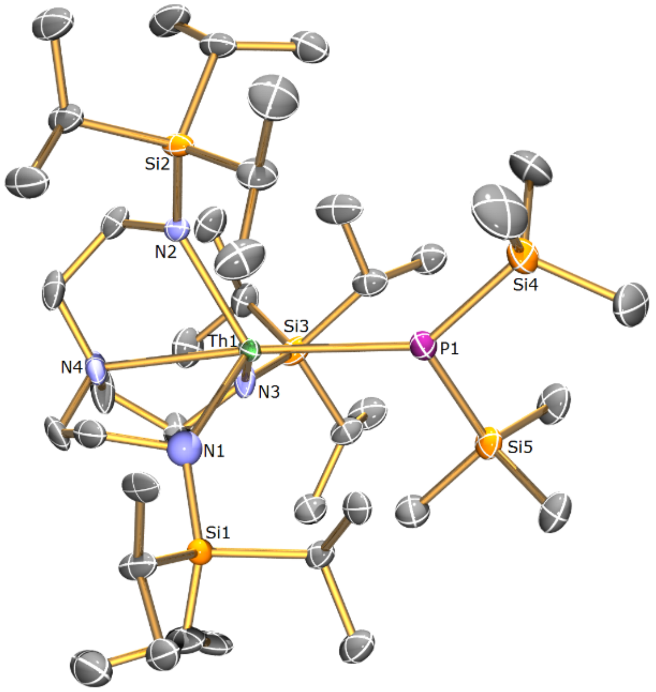

Figure S15. Solid state structure of **4ThP** at 150 K and displacement ellipsoids set to 40% and hydrogen atoms, minor disorder components, and any lattice solvent omitted for clarity.

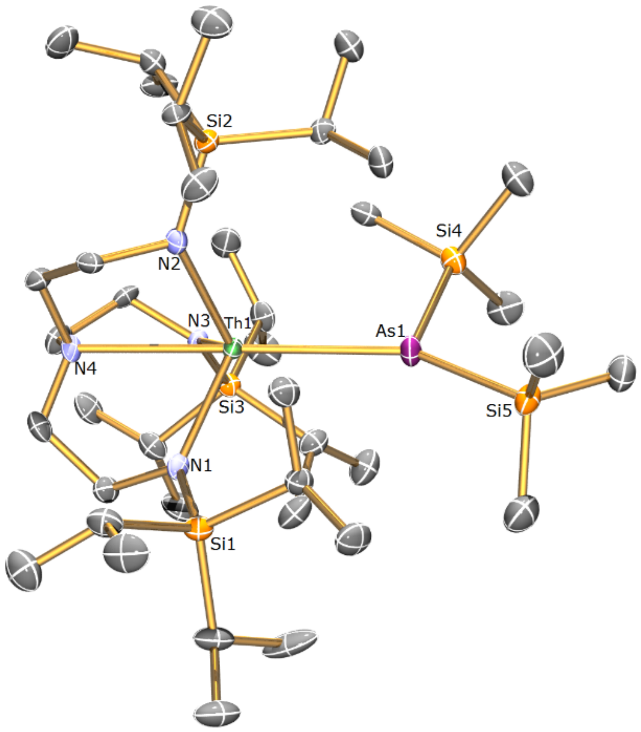

Figure S16. Solid state structure of **4ThAs** at 150 K and displacement ellipsoids set to 40% and hydrogen atoms, minor disorder components, and any lattice solvent omitted for clarity.

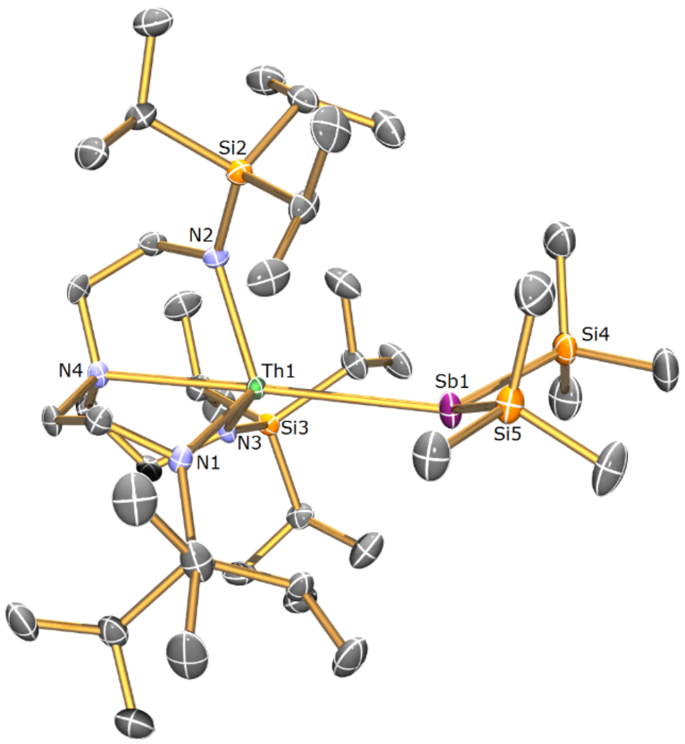

Figure S17. Solid state structure of **4ThSb** at 150 K and displacement ellipsoids set to 40% and hydrogen atoms, minor disorder components, and any lattice solvent omitted for clarity.

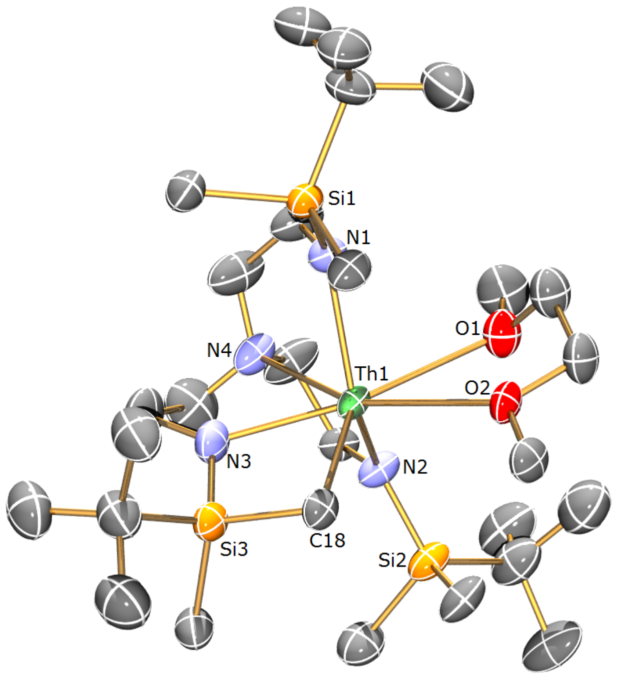

Figure S18. Solid state structure of **5** at 150 K and displacement ellipsoids set to 40% and hydrogen atoms, minor disorder components, and any lattice solvent omitted for clarity.

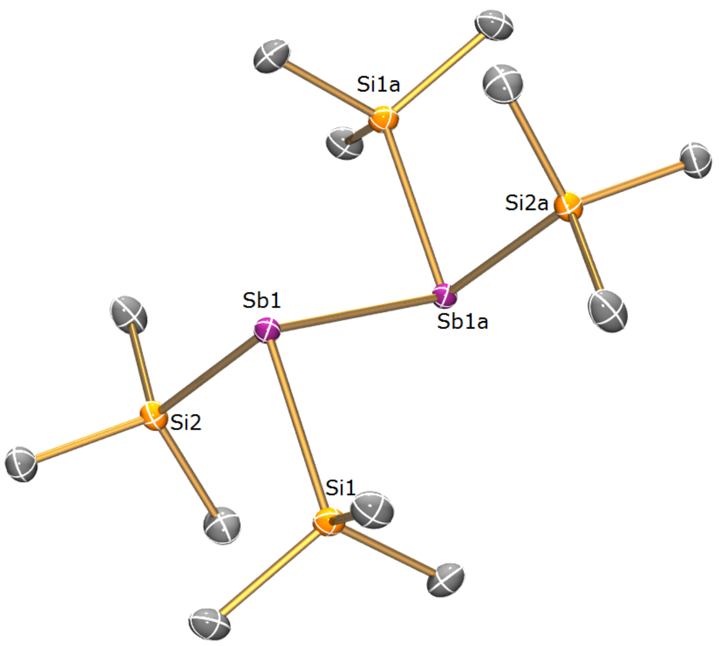

Figure S19. Solid state structure of **Sb_2** at 150 K and displacement ellipsoids set to 40% and hydrogen atoms, minor disorder components, and any lattice solvent omitted for clarity.

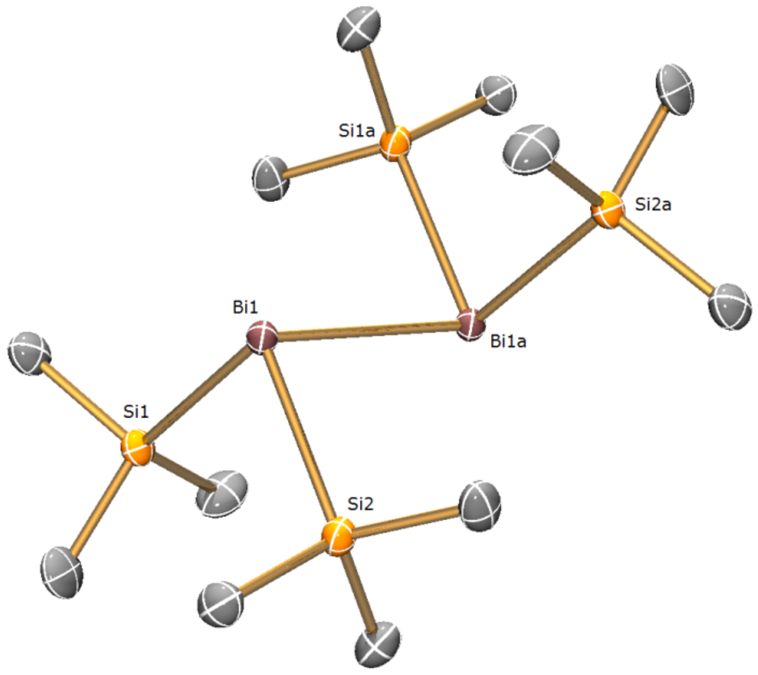

Figure S20. Solid state structure of **Bi_2** at 150 K and displacement ellipsoids set to 40% and hydrogen atoms, minor disorder components, and any lattice solvent omitted for clarity.

Computational Details

General

Unrestricted (uranium) and restricted (thorium) geometry optimisations were performed for the full models of **3UP**, **3UAs**, **3USb**, **3UBi**, **3ThP**, **3ThAs**, **4UP**, **4UAs**, **4USb**, **4ThP**, **4ThAs**, and **4ThSb** using coordinates derived from the X-ray crystal structures. No constraints were imposed on the calculations. The calculations were performed using the Amsterdam Density Functional (ADF) suite version 2012.01.[7,8] The DFT geometry optimisations employed Slater type orbital (STO) triple-ζ-plus polarisation all-electron basis sets (from the ZORA/TZP database of the ADF suite). Scalar relativistic approaches were used within the ZORA Hamiltonian for the inclusion of relativistic effects and the local density approximation (LDA) with the correlation potential due to Vosko et al[9] was used in all of the calculations. Gradient corrections were performed using the functionals of Becke[10] and Perdew.[11] MOLEKEL[12] was used to prepare the three-dimensional plot of the electron density. Natural Bond Order (NBO) analyses were carried out with NBO 5.0.[13] The Atoms in Molecules analysis[14,15] was carried out with Xaim-1.0.[16]

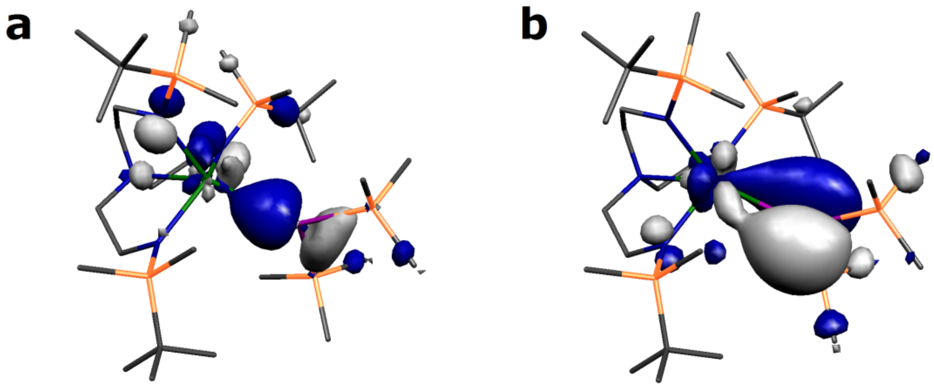

Figure S21. Kohn–Sham molecular orbital representations of the principal U–P interactions of **3UP**. HOMO−6 (a, −5.480 eV) and HOMO–2 (b, −3.964 eV).

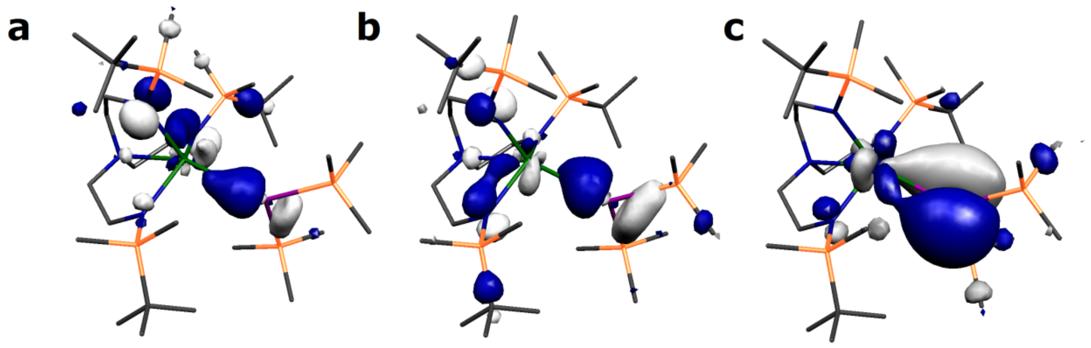

Figure S22. Kohn–Sham molecular orbital representations of the principal U–As interactions of **3UAs**. HOMO−6 (a, −5.425 eV), HOMO–5 (b, −5.254 eV) and HOMO–2 (c, −3.768 eV).

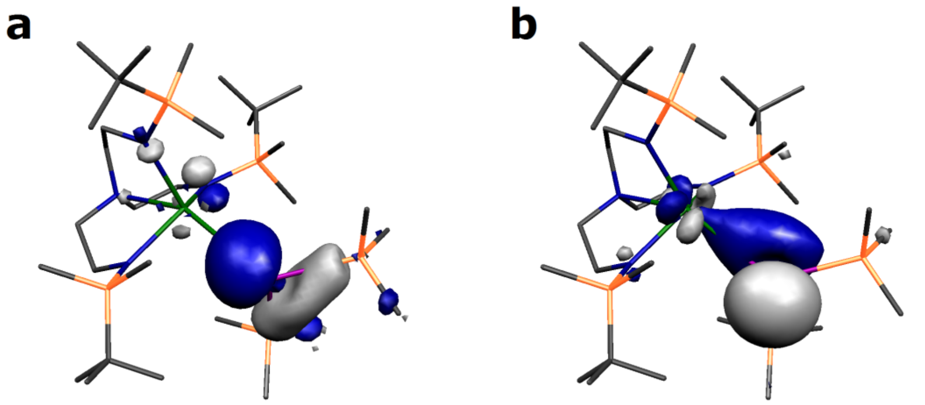

Figure S23. Kohn–Sham molecular orbital representations of the principal U–Sb interactions of **3USb**. HOMO−3 (a, −4.854 eV) and HOMO–2 (b, −3.624 eV).

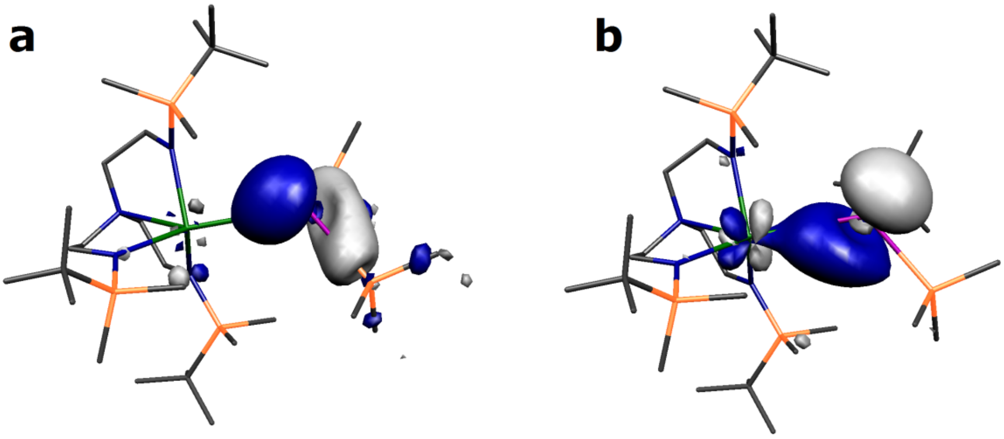

Figure S24. Kohn–Sham molecular orbital representations of the principal U–Bi interactions of **3UBi**. HOMO−3 (a, −4.732 eV) and HOMO–2 (b, −3.561 eV).

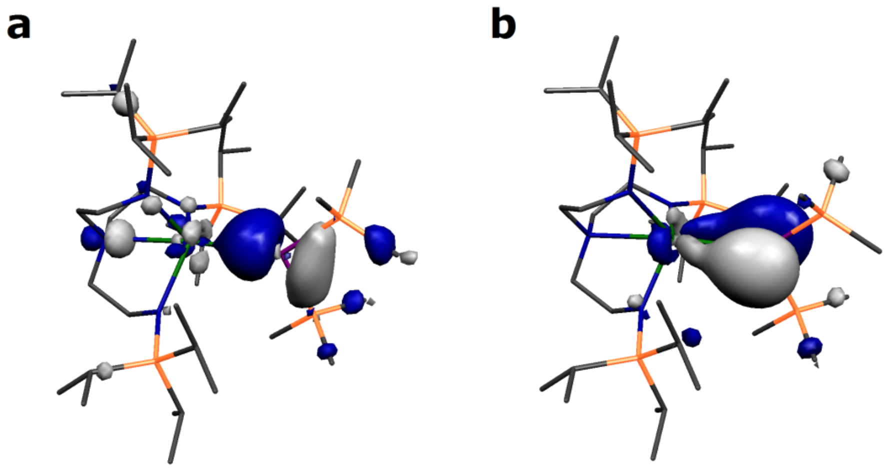

Figure S25. Kohn–Sham molecular orbital representations of the principal U–P interactions of **4UP**. HOMO−6 (a, −5.545 eV) and HOMO–2 (b, −4.121 eV).

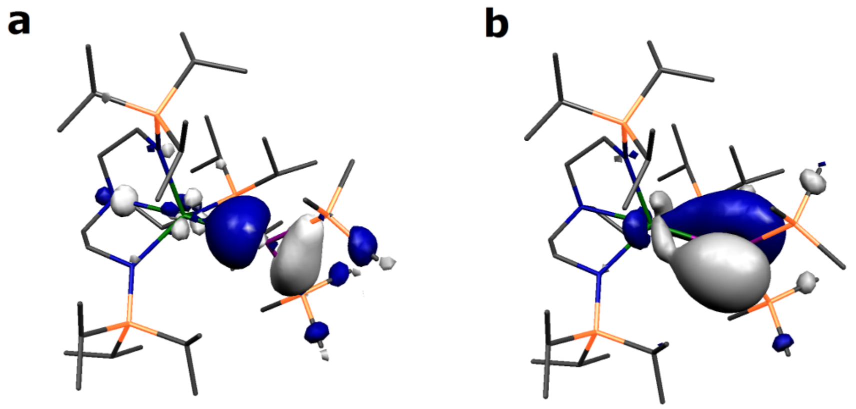

Figure S26. Kohn–Sham molecular orbital representations of the principal U–As interactions of **4UAs**. HOMO−6 (a, −5.510 eV) and HOMO–2 (b, −3.881 eV).

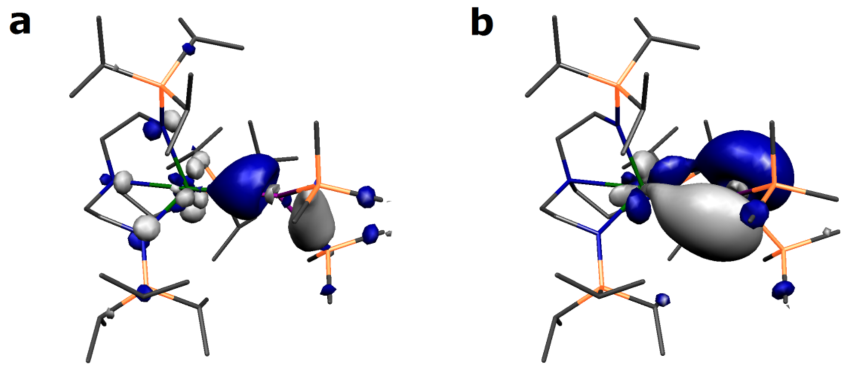

Figure S27. Kohn–Sham molecular orbital representations of the principal U–Sb interactions of **4USb**. HOMO−6 (a, −5.342 eV) and HOMO–2 (b, −3.682 eV).

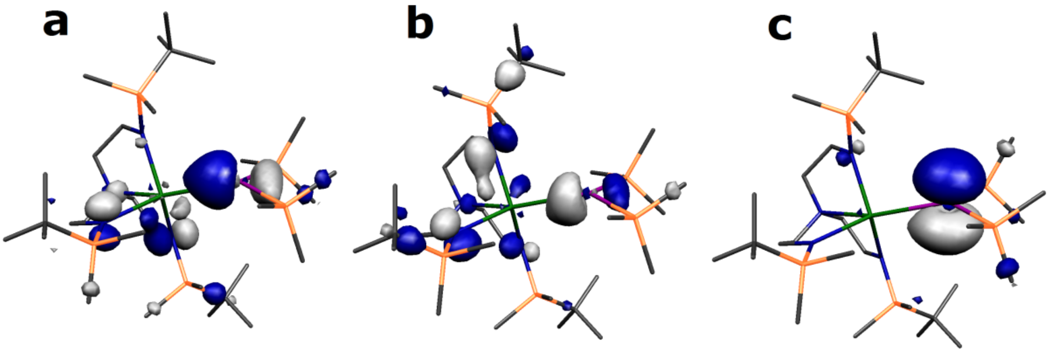

Figure S28. Kohn–Sham molecular orbital representations of the principal Th–P interactions of **3ThP**. HOMO−4 (a, −5.441 eV), HOMO−3 (b, −5.258 eV) and HOMO (c, −3.927 eV).

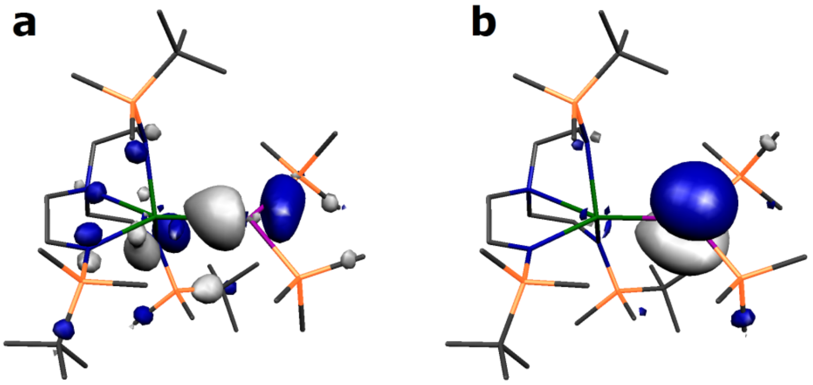

Figure S29. Kohn–Sham molecular orbital representations of the principal Th–As interactions of **3ThAs**. HOMO−4 (a, −5.354 eV) and HOMO (b, −3.727 eV).

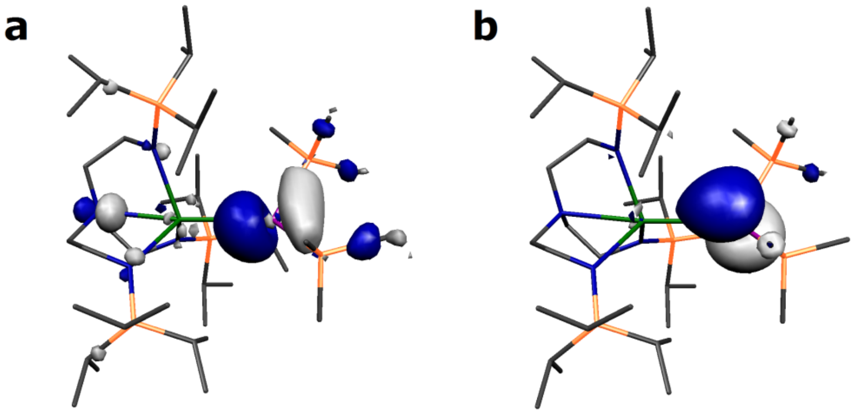

Figure S30. Kohn–Sham molecular orbital representations of the principal Th–P interactions of **4ThP**. HOMO−4 (a, − 5.558 eV) and HOMO (b, −4.036 eV).

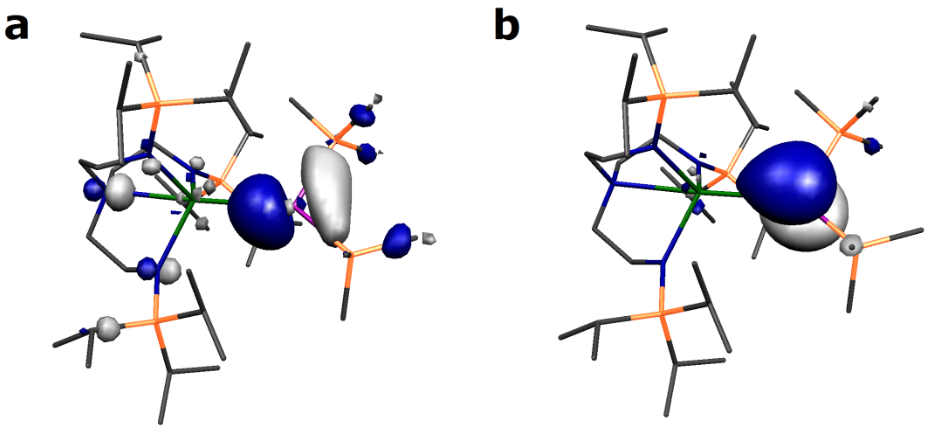

Figure S31. Kohn–Sham molecular orbital representations of the principal Th–As interactions of **4ThAs**. HOMO−4 (a, −5.446 eV) and HOMO (b, −3.831 eV).

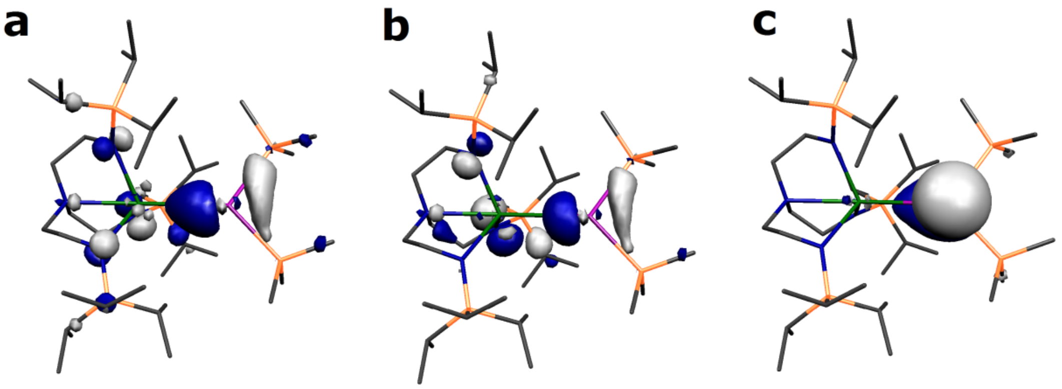

Figure S32. Kohn–Sham molecular orbital representations of the principal Th–Sb interactions of **4ThSb**. HOMO−4 (a, −5.271 eV), HOMO–1 (b, −4.977 eV) and HOMO (c, −3.588 eV).

Table S1. Final Coordinates and Single Point Energy of 3UP After Geometry Optimisation

1.C	3.274536	2.227366	-4.628646
2.C	0.138621	0.788506	-3.514603
3.C	-1.213941	0.074518	-3.445905
4.C	3.512898	1.876913	-3.142398
5.C	3.896493	0.390988	-3.023745
6.C	1.209151	3.901371	-2.600393
7.C	4.686663	2.737811	-2.619750
8.C	-2.293547	1.917643	-2.191813
9.C	-3.147667	-0.393151	-2.030559
10.C	1.519684	-2.925319	-1.964438
11.C	-2.718251	-1.728496	-1.433711
12.C	4.466458	-2.739163	-1.011561
13.C	-2.574345	2.387476	-0.760749
14.C	-3.187890	-4.840860	-0.043851
15.C	2.407909	2.376618	-0.243753
16.C	2.629176	-4.746507	0.252076
17.C	-0.929168	-4.640958	1.020416
18.C	-2.409659	-4.241426	1.148548
19.C	-0.636601	4.631357	1.216427
20.C	-4.380983	-1.856907	1.433783
21.C	-3.897127	4.221180	1.975901
22.C	4.902928	0.053306	1.983733
23.C	-2.983030	-4.838130	2.454536
24.C	-2.972154	3.192772	2.667794
25.C	-3.731520	1.860577	2.823759
26.C	4.287143	-2.747906	3.030344
27.C	-0.088570	1.968153	2.711906
28.C	-1.608751	-1.669382	2.727512
29.C	-2.610069	3.729259	4.072087
30.C	2.687279	-0.343817	4.066231
31.H	4.178082	1.992497	-5.219550
32.H	2.442689	1.656539	-5.067680
33.H	3.059925	3.297240	-4.771310
34.H	-1.813015	0.265471	-4.358450
35.H	0.788811	0.199677	-4.188127
36.H	0.004227	1.765149	-4.016861
37.H	0.828638	3.906992	-3.632749
38.H	4.849633	0.191564	-3.545388
39.H	3.132333	-0.267215	-3.462225
40.H	-1.054570	-1.012724	-3.385478
41.H	5.572783	2.598158	-3.264831
42.H	-3.697777	-0.519500	-2.984626
43.H	-3.161240	2.132320	-2.846354
44.H	1.977916	4.687970	-2.538072
45.H	1.779038	-3.713052	-2.689558
46.H	-1.427659	2.466640	-2.577828
47.H	4.450490	3.814187	-2.619151
48.H	1.596471	-1.952877	-2.475151
49.H	0.385687	4.192538	-1.931075
50.H	4.633809	-3.289624	-1.953455
51.H	4.019749	0.089829	-1.974329
52.H	-2.174584	-2.325427	-2.189197
53.H	4.979057	2.455424	-1.597300
54.H	0.469716	-3.079322	-1.668031

55.H	-3.814782	0.106284	-1.316003
56.H	-3.635519	-2.303772	-1.224656
57.H	-2.775873	-4.517054	-1.010476
58.H	4.732525	-1.688290	-1.184457
59.H	-2.658262	3.489424	-0.811116
60.H	2.995017	-5.445325	-0.519650
61.H	5.164818	-3.147675	-0.264585
62.H	-3.574318	2.030583	-0.449603
63.H	-4.257000	-4.577743	-0.022009
64.H	-3.124778	-5.943897	-0.018559
65.H	3.165337	3.162272	-0.104194
66.H	2.807109	1.435983	0.166424
67.H	1.623883	-5.066492	0.553616
68.H	-0.491626	-4.251272	0.091001
69.H	1.537997	2.678530	0.360957
70.H	-1.258808	5.152823	0.472688
71.H	-5.040855	-2.304430	0.674870
72.H	-0.823610	-5.741120	1.007274
73.H	3.290136	-4.839286	1.126160
74.H	0.376816	4.550169	0.796775
75.H	-4.234603	3.880834	0.985893
76.H	5.423560	-0.335721	1.096004
77.H	-4.521900	-0.766301	1.404144
78.H	4.536989	1.062711	1.743931
79.H	-3.406952	5.197790	1.846050
80.H	-0.321202	-4.256435	1.852214
81.H	-4.006124	1.430558	1.849736
82.H	-0.578313	5.279293	2.105293
83.H	4.830365	-3.216082	2.196855
84.H	-2.890814	-5.939206	2.436856
85.H	-4.050376	-4.602236	2.587196
86.H	-4.801745	4.387071	2.588209
87.H	0.720689	1.487893	2.129951
88.H	-4.737401	-2.205711	2.416201
89.H	5.643559	0.148218	2.795537
90.H	-0.518866	-1.798605	2.630030
91.H	-1.819007	-0.601485	2.889254
92.H	-2.444513	-4.481698	3.345736
93.H	-4.662491	2.009668	3.399301
94.H	3.537331	-3.465508	3.393712
95.H	-0.555750	1.182398	3.321795
96.H	-3.137483	1.106441	3.360351
97.H	5.012725	-2.570979	3.841445
98.H	0.423107	2.655745	3.402888
99.H	-1.938645	-2.200241	3.633557
100.H	-2.073584	4.690047	4.025587
101.H	2.244647	0.640596	3.856317
102.H	1.890608	-0.993066	4.458476
103.H	-3.530359	3.897735	4.659870
104.H	-1.985501	3.021118	4.637797
105.H	3.441758	-0.211243	4.858684
106.N	-1.956887	0.471016	-2.225312
107.N	0.718883	0.944007	-2.172020
108.N	-1.889160	-1.502891	-0.229444
109.N	-1.542903	1.947677	0.198440

110.P	1.881839	-1.380277	0.954789
111.Si	1.938289	2.223841	-2.069048
112.Si	2.663143	-2.977128	-0.440146
113.Si	-2.540436	-2.307184	1.210945
114.Si	-1.344478	2.918972	1.647949
115.Si	3.487438	-1.100080	2.520170
116.U	-0.269261	0.117377	-0.281638

Energy: −616.08879950 eV

Table S2. Final Coordinates and Single Point Energy of 3UAs After Geometry Optimisation

1.C	-0.244545	-1.003499	-5.934179
2.C	2.146418	-0.344950	-5.560023
3.C	0.851442	-0.819681	-4.860626
4.C	-0.401001	1.999140	-4.416897
5.C	1.124384	-2.168278	-4.169890
6.C	-2.127631	-0.481719	-2.857456
7.C	1.857044	1.071826	-2.577843
8.C	2.484682	-4.593834	-1.661306
9.C	-3.177077	-0.507305	-1.742281
10.C	5.006636	-1.371126	-1.389602
11.C	-2.970638	1.914063	-1.271883
12.C	0.800907	4.692292	-1.092719
13.C	-0.402625	-3.945340	-0.739115
14.C	-2.203537	2.902068	-0.386761
15.C	-3.748465	0.348442	0.472101
16.C	5.038188	-3.114813	1.133353
17.C	1.684642	-5.146537	1.191634
18.C	1.863696	2.627944	0.965045
19.C	-1.257176	5.828341	1.360823
20.C	4.797023	-0.063938	1.382503
21.C	-3.192928	-0.787068	1.323209
22.C	-0.202945	4.806222	1.846274
23.C	1.031050	5.585615	2.356152
24.C	-0.796442	3.989004	3.011021
25.C	-0.526830	-3.294670	3.463188
26.C	0.346228	0.068977	3.626095
27.C	-2.959817	-2.959581	3.960832
28.C	-1.547408	-2.357568	4.133980
29.C	-2.606497	0.561523	4.275992
30.C	-1.237048	-2.274408	5.646433
31.H	0.073152	-1.762607	-6.670498
32.H	2.417394	-1.048919	-6.367087
33.H	-0.440099	-0.073372	-6.488809
34.H	-1.198071	-1.345903	-5.506928
35.H	2.035284	0.649909	-6.019851
36.H	0.301548	2.370510	-5.180141
37.H	2.997226	-0.303526	-4.864137
38.H	-1.350048	1.788529	-4.931523
39.H	1.549302	-2.893385	-4.886317
40.H	0.205818	-2.608514	-3.756195
41.H	-0.575017	2.819016	-3.703357
42.H	-2.447510	0.235329	-3.636818
43.H	-2.141926	-1.473167	-3.346599
44.H	1.833141	-2.060381	-3.336920

45.H	2.611958	1.470629	-3.271227
46.H	2.478663	-3.949210	-2.549102
47.H	2.093235	-5.582302	-1.955292
48.H	-2.509318	1.900458	-2.265587
49.H	-4.199739	-0.394989	-2.153474
50.H	4.727779	-2.211684	-2.041912
51.H	2.323186	0.262403	-1.992665
52.H	4.664575	-0.443310	-1.871698
53.H	1.207170	4.079888	-1.911039
54.H	1.599465	1.896831	-1.896266
55.H	6.107684	-1.335060	-1.331791
56.H	3.530122	-4.734754	-1.348977
57.H	-0.581169	-3.279628	-1.596956
58.H	-4.026219	2.231590	-1.382960
59.H	-0.071987	5.232889	-1.490226
60.H	-3.141530	-1.477824	-1.225244
61.H	-0.717696	-4.962937	-1.021927
62.H	-2.170966	3.856305	-0.945869
63.H	1.561078	5.445882	-0.833052
64.H	-1.046349	-3.614437	0.090436
65.H	-4.801856	0.171986	0.175534
66.H	4.875514	-4.011087	0.517767
67.H	-0.880139	6.452642	0.537083
68.H	2.144441	1.821716	0.263085
69.H	-2.798084	3.113733	0.521993
70.H	1.382740	-6.155245	0.861124
71.H	4.379338	0.874235	0.988102
72.H	-3.709933	1.273477	1.062148
73.H	-3.353012	-1.755559	0.815110
74.H	-2.183582	5.345263	1.018312
75.H	6.127279	-2.975772	1.237016
76.H	5.895707	0.027761	1.376402
77.H	2.742767	-5.193064	1.488879
78.H	2.745103	3.285131	1.019935
79.H	1.499710	6.187171	1.561699
80.H	4.621750	-3.309524	2.131904
81.H	-1.530806	6.507842	2.187744
82.H	1.096763	-4.887977	2.081669
83.H	1.740430	2.171877	1.958074
84.H	4.469076	-0.163724	2.427884
85.H	-3.809088	-0.842557	2.236109
86.H	1.800181	4.917012	2.771589
87.H	-0.723673	-3.394459	2.386844
88.H	-1.670377	3.401447	2.694266
89.H	-3.214706	-3.117602	2.903043
90.H	0.732931	6.282660	3.159765
91.H	1.097550	-0.467685	3.024698
92.H	-0.064832	3.286776	3.436639
93.H	0.399911	1.140097	3.381211
94.H	-1.118826	4.657048	3.829445
95.H	0.505459	-2.931282	3.571023
96.H	-0.576616	-4.303820	3.910942
97.H	-2.622605	1.562255	3.818802
98.H	-3.742736	-2.329079	4.410572
99.H	-3.012269	-3.945213	4.457833

100.H	-3.641091	0.188391	4.308498
101.H	0.624821	-0.029495	4.686337
102.H	-2.276358	0.684755	5.319823
103.H	-0.227579	-1.882388	5.843441
104.H	-1.288181	-3.282780	6.096008
105.H	-1.957957	-1.640188	6.185292
106.As	1.897515	-1.662737	0.392240
107.N	-0.801326	-0.144297	-2.319080
108.N	-2.892556	0.534929	-0.725910
109.N	-0.858380	2.413977	-0.029212
110.N	-1.758396	-0.556085	1.605364
111.Si	0.330945	0.482007	-3.527104
112.Si	1.434070	-3.906750	-0.228262
113.Si	4.260981	-1.565275	0.349885
114.Si	0.340419	3.620365	0.410915
115.Si	-1.403771	-0.591055	3.343230
116.U	-0.401355	0.183016	-0.095319

Energy: –615.33981340 eV

Table S3. Final Coordinates and Single Point Energy of 3USb After Geometry Optimisation

1.C	0.712561	2.706874	-5.350870
2.C	3.011105	3.032296	-4.394613
3.C	1.867403	-0.313599	-4.293912
4.C	1.530271	2.760190	-4.039917
5.C	-0.187062	-3.956289	-3.319861
6.C	1.006343	3.919763	-3.169723
7.C	-1.351485	0.757749	-3.185760
8.C	-2.398711	-0.256786	-2.712634
9.C	1.413059	-5.439999	-2.091049
10.C	0.084787	-4.652711	-1.971099
11.C	-1.041845	-5.669128	-1.673412
12.C	2.660489	1.112816	-1.645074
13.C	-3.480836	1.059382	-0.915578
14.C	-2.299604	-2.530450	-0.731043
15.C	1.957285	-2.491117	-0.756327
16.C	-3.268568	-1.352515	-0.670200
17.C	-2.077867	4.507915	-0.319970
18.C	-3.152711	1.503924	0.506541
19.C	0.647986	3.506106	0.660238
20.C	0.310477	-4.321013	1.112779
21.C	4.909003	-0.590703	1.672028
22.C	3.681216	-3.203541	2.721483
23.C	-1.210462	5.477651	2.859769
24.C	-1.641426	3.998025	2.727954
25.C	-3.153696	3.898007	3.029433
26.C	-1.894716	-1.276544	3.396536
27.C	-0.878298	3.154918	3.767688
28.C	4.340242	-0.961162	4.686442
29.C	0.300216	-2.908815	4.846554
30.C	-0.416420	-0.133455	5.836937
31.H	0.839693	3.649253	-5.913239
32.H	1.036880	1.887066	-6.009953
33.H	-0.364471	2.584167	-5.163886
34.H	3.097252	3.985839	-4.946117
35.H	3.437882	2.246060	-5.036911

36.H	2.748518	-0.058103	-4.903028
37.H	1.040086	-0.533020	-4.986525
38.H	-0.204435	-4.698483	-4.138179
39.H	-1.129890	0.502876	-4.238570
40.H	2.096594	-1.245247	-3.755314
41.H	1.043388	4.870060	-3.731554
42.H	3.644325	3.115442	-3.497745
43.H	0.587714	-3.215294	-3.564989
44.H	-1.156936	-3.437433	-3.333296
45.H	-3.338190	-0.145184	-3.289167
46.H	-1.801341	1.767755	-3.215222
47.H	1.305697	-6.244961	-2.839866
48.H	-2.005324	-1.265262	-2.887880
49.H	-0.034616	3.759318	-2.854008
50.H	-1.104777	-6.413609	-2.487527
51.H	2.245840	-4.798603	-2.415180
52.H	1.610304	4.059057	-2.261257
53.H	3.656595	0.818542	-2.010893
54.H	-2.388682	-3.015194	-1.720264
55.H	-2.030037	-5.195333	-1.587368
56.H	-3.209717	1.870426	-1.603467
57.H	2.040567	-2.002398	-1.737510
58.H	1.704277	-5.915865	-1.141665
59.H	-1.820451	4.199952	-1.344778
60.H	-4.223474	-1.597239	-1.176399
61.H	2.760142	2.113420	-1.201058
62.H	-4.558179	0.838415	-1.050963
63.H	-0.855784	-6.221524	-0.739940
64.H	2.452037	0.413101	-0.812374
65.H	2.793031	-3.200267	-0.669822
66.H	-3.175046	4.504790	-0.234056
67.H	-1.746524	5.550790	-0.197978
68.H	0.915791	3.132098	-0.339943
69.H	-2.650782	-3.285008	-0.001124
70.H	2.114342	-1.738196	0.038665
71.H	-3.503147	-1.123552	0.379343
72.H	0.959406	4.561618	0.690975
73.H	4.622595	-0.852558	0.642097
74.H	-3.729280	2.420109	0.714769
75.H	0.947888	-5.218499	1.083862
76.H	-3.528279	0.761251	1.233229
77.H	-0.703074	-4.636552	1.405104
78.H	1.236259	2.952970	1.408303
79.H	4.958020	0.506412	1.739126
80.H	3.393339	-3.501535	1.703536
81.H	5.921648	-0.988570	1.848296
82.H	0.694556	-3.654998	1.898870
83.H	-1.769908	6.135950	2.176925
84.H	-3.766590	4.455690	2.304012
85.H	-0.136495	5.617480	2.661849
86.H	-1.838047	-1.934501	2.515448
87.H	4.694445	-3.594254	2.914472
88.H	-2.217176	-0.279931	3.056546
89.H	-3.500880	2.854371	3.040977
90.H	2.992439	-3.695362	3.422853

91.H	-1.401352	5.834727	3.887852
92.H	0.212082	3.217183	3.644009
93.H	-3.367421	4.321237	4.027420
94.H	-1.148644	2.092486	3.696707
95.H	-2.669753	-1.671003	4.074401
96.H	0.367628	-3.633322	4.023473
97.H	5.338547	-1.411879	4.816461
98.H	-1.117624	3.497799	4.790399
99.H	4.421742	0.119360	4.874315
100.H	1.268258	-2.894674	5.367975
101.H	3.677923	-1.384647	5.455949
102.H	-0.456560	-3.277974	5.559317
103.H	-0.746513	0.891216	5.616832
104.H	0.525188	-0.066004	6.401695
105.H	-1.172220	-0.604261	6.489115
106.N	-0.146155	0.746879	-2.338358
107.N	-2.658811	-0.131504	-1.254218
108.N	-0.922676	-2.089642	-0.470611
109.N	-1.692344	1.692087	0.639537
110.Sb	1.447798	-0.035049	2.581594
111.Si	1.409500	1.079477	-3.081560
112.Si	0.296379	-3.372391	-0.523280
113.Si	-1.217692	3.368648	0.945459
114.Si	3.681839	-1.311938	2.936513
115.Si	-0.195309	-1.168163	4.256707
116.U	-0.297865	0.082452	-0.174865

Energy: −614.17269560 eV

Table S4. Final Coordinates and Single Point Energy of 3UBi After Geometry Optimisation

1.C	3.335064	2.230800	-4.034031
2.C	0.303779	1.736245	-3.705252
3.C	1.475075	4.556800	-3.411242
4.C	1.776243	-4.746236	-3.173520
5.C	2.940073	-1.607912	-2.946114
6.C	-0.638532	-2.252298	-2.572750
7.C	-2.019527	-1.606207	-2.496303
8.C	1.978899	-4.291181	-1.709293
9.C	3.392236	-4.741465	-1.265076
10.C	-3.686870	4.452845	-1.115191
11.C	-3.133670	1.011743	-1.291477
12.C	5.476582	0.579055	-1.067893
13.C	-3.585040	-0.426418	-1.054918
14.C	0.945009	-4.986028	-0.800054
15.C	-1.263297	4.783782	-0.577701
16.C	5.512650	3.521444	-0.248986
17.C	-2.584628	-2.553946	-0.278479
18.C	-2.632058	4.393501	0.012523
19.C	2.629361	-1.858474	0.121264
20.C	-3.023458	5.413862	1.107379
21.C	-2.477867	-2.257159	1.221623
22.C	-4.201904	2.209373	1.592590
23.C	4.795243	1.352422	1.825754
24.C	-1.231002	2.652555	2.195837
25.C	0.279117	-3.478777	3.135495
26.C	0.939482	-0.434147	3.133766

27.C	-2.332034	-0.160422	4.581735
28.C	-1.568399	-1.497812	4.648066
29.C	-2.587552	-2.653282	4.776464
30.C	-0.664830	-1.496169	5.903209
31.H	3.171438	2.485571	-5.094722
32.H	0.162375	1.906225	-4.785906
33.H	1.379232	4.703515	-4.500934
34.H	3.551288	1.155091	-3.976585
35.H	2.543332	-4.322883	-3.839308
36.H	2.442779	-1.708599	-3.922546
37.H	4.226723	2.775991	-3.692689
38.H	0.793836	-4.461852	-3.577230
39.H	1.853072	-5.846260	-3.242488
40.H	-0.272340	-2.126397	-3.609483
41.H	0.444416	0.657080	-3.538801
42.H	2.303240	5.186409	-3.053285
43.H	0.552719	4.920033	-2.936200
44.H	3.937547	-2.066305	-3.027904
45.H	-0.620651	2.041837	-3.190696
46.H	-2.002807	-0.642430	-3.025357
47.H	-2.782400	-2.240671	-2.989259
48.H	3.075555	-0.535720	-2.747097
49.H	-0.746256	-3.342620	-2.429377
50.H	-2.794685	1.137599	-2.335320
51.H	5.406101	0.817160	-2.139208
52.H	-3.414840	3.810447	-1.965756
53.H	4.188882	-4.241497	-1.837609
54.H	-4.341864	-0.757104	-1.793572
55.H	3.507721	-5.828262	-1.426040
56.H	-3.766612	5.484749	-1.502057
57.H	5.460779	3.849350	-1.298024
58.H	-0.950031	4.078753	-1.360598
59.H	-4.020261	1.660687	-1.201339
60.H	6.545947	0.552209	-0.796823
61.H	1.060106	-6.083523	-0.859038
62.H	-0.090607	-4.753605	-1.089609
63.H	-1.312281	5.790851	-1.029600
64.H	-4.690436	4.158724	-0.769878
65.H	5.064752	-0.429174	-0.919473
66.H	3.571571	-4.547029	-0.197526
67.H	-1.779832	-3.247181	-0.550743
68.H	-3.553335	-3.036479	-0.514188
69.H	6.577344	3.411426	0.018657
70.H	-4.033578	-0.489062	-0.055238
71.H	5.081708	4.321232	0.370892
72.H	-0.465801	4.800803	0.179325
73.H	1.065832	-4.698812	0.254806
74.H	3.690004	-2.140012	0.190477
75.H	2.596359	-0.760199	0.255441
76.H	-3.036414	6.434455	0.684240
77.H	-5.031956	2.211660	0.869935
78.H	2.107868	-2.338733	0.962054
79.H	-4.026946	5.217845	1.517025
80.H	-3.390740	-1.734118	1.562420
81.H	-2.310027	5.419137	1.946126

82.H	-2.489918	-3.236902	1.733513
83.H	5.863663	1.295154	2.091837
84.H	-0.244464	3.013341	1.866872
85.H	-4.168394	1.214779	2.062514
86.H	4.350686	0.362608	2.009441
87.H	-4.456532	2.935950	2.379634
88.H	4.314704	2.069062	2.508724
89.H	1.345782	-0.093927	2.159092
90.H	-1.106768	1.647809	2.628416
91.H	1.126474	-3.572499	2.439460
92.H	-0.463919	-4.239422	2.848346
93.H	-1.573635	3.302748	3.015259
94.H	1.811116	-0.854754	3.659902
95.H	-2.970213	-0.095144	3.688741
96.H	0.637997	0.473565	3.676259
97.H	-3.298641	-2.674861	3.937437
98.H	0.646073	-3.738445	4.141140
99.H	-1.651352	0.702932	4.565764
100.H	-2.095742	-3.636435	4.828675
101.H	-2.981324	-0.043573	5.467492
102.H	-3.179572	-2.533157	5.701282
103.H	-0.105174	-2.437806	6.015675
104.H	0.064003	-0.670841	5.889680
105.H	-1.281345	-1.373539	6.811646
106.Bi	1.951314	2.340547	-0.349753
107.N	0.271054	-1.666311	-1.578505
108.N	-2.399166	-1.321718	-1.089187
109.N	-2.060390	1.356426	-0.334269
110.N	-1.274513	-1.470072	1.540317
111.Si	1.794103	2.719338	-3.027864
112.Si	1.900529	-2.354972	-1.554734
113.Si	4.586853	1.876925	0.004514
114.Si	-2.515845	2.630178	0.805270
115.Si	-0.462189	-1.728056	3.074356
116.U	-0.350292	-0.119707	-0.023230

Energy: −613.55688129 eV

Table S5. Final Coordinates and Single Point Energy of 4UP After Geometry Optimisation

1.C	-0.232900	-1.337475	-5.897086
2.C	3.489645	-0.731935	-5.476165
3.C	1.017650	-3.391028	-5.174532
4.C	2.377339	1.409333	-4.758915
5.C	0.328970	-2.100402	-4.676668
6.C	2.780923	-0.008842	-4.309705
7.C	-1.952217	0.377029	-3.155419
8.C	-0.514503	0.880954	-3.192577
9.C	3.592722	-2.915192	-2.681916
10.C	2.308099	-2.223526	-2.184413
11.C	-0.221512	4.646639	-1.569095
12.C	1.377712	-3.247789	-1.512661
13.C	-3.255801	-0.861781	-1.467953
14.C	-3.074162	1.591917	-1.328466
15.C	3.223587	2.011260	-1.104110
16.C	-2.363190	-2.014710	-1.017263
17.C	-2.457672	5.429395	-0.689599

18.C	5.349512	-0.071786	-0.380780
19.C	-1.037862	4.965718	-0.304609
20.C	-2.816195	1.844490	0.151813
21.C	-5.103534	-2.671973	1.095077
22.C	-0.576072	-4.830151	1.271479
23.C	-2.042708	-4.382174	1.416255
24.C	4.746786	2.245887	1.522078
25.C	1.632454	4.873014	1.570747
26.C	0.881801	3.530313	1.686011
27.C	-4.074170	-2.013996	2.040916
28.C	-2.780465	-5.289599	2.423823
29.C	-2.193677	4.135617	2.415266
30.C	-1.794681	5.478469	3.065844
31.C	5.014533	-1.165334	3.198851
32.C	-1.083827	-1.798722	3.093479
33.C	2.662687	-3.071296	3.098449
34.C	1.019642	2.972494	3.115067
35.C	-4.526569	-2.240128	3.500776
36.C	-1.399248	-0.330153	3.431790
37.C	-2.527736	3.085017	3.489763
38.C	-1.030729	-2.649153	4.377722
39.C	2.448206	-0.430464	4.656826
40.H	-0.869049	-2.002772	-6.506538
41.H	0.574821	-0.979295	-6.553228
42.H	2.823211	-0.839034	-6.346066
43.H	4.365294	-0.150769	-5.813057
44.H	0.329224	-3.968269	-5.816196
45.H	1.911266	-3.170590	-5.777461
46.H	-0.844361	-0.466014	-5.627157
47.H	1.647080	1.385380	-5.583104
48.H	3.846850	-1.735413	-5.207924
49.H	3.259451	1.957481	-5.131891
50.H	1.326270	-4.048924	-4.351146
51.H	-0.534579	-2.422025	-4.061093
52.H	-0.198652	0.990489	-4.241049
53.H	1.947126	2.001533	-3.940709
54.H	-2.633311	1.000111	-3.765835
55.H	3.529623	0.119021	-3.504117
56.H	-1.976100	-0.642343	-3.561684
57.H	3.391634	-3.651626	-3.474233
58.H	4.328832	-2.199949	-3.074208
59.H	-0.485781	1.907317	-2.783308
60.H	-0.148488	5.525447	-2.232056
61.H	-3.862535	-1.122231	-2.355933
62.H	1.003700	-4.002956	-2.220346
63.H	-0.697106	3.842704	-2.151838
64.H	4.076224	-3.456894	-1.851879
65.H	-2.629886	2.414717	-1.904528
66.H	-1.660205	-2.261007	-1.833849
67.H	4.034907	2.391998	-1.744174
68.H	2.550419	1.416099	-1.737647
69.H	-4.156723	1.559992	-1.562287
70.H	-2.414412	6.264814	-1.409490
71.H	0.803210	4.317831	-1.343453
72.H	2.618594	-1.524781	-1.380997

73.H	4.975234	-0.776007	-1.137858
74.H	-3.040725	4.627426	-1.169674
75.H	0.496002	-2.779728	-1.044688
76.H	-2.981102	-2.919275	-0.891555
77.H	6.111179	0.569742	-0.854945
78.H	-3.946117	-0.612060	-0.652567
79.H	1.910240	-3.784644	-0.711616
80.H	2.663186	2.881547	-0.733839
81.H	-0.548166	5.826949	0.188841
82.H	-3.032565	5.780202	0.179611
83.H	-4.927864	-2.449592	0.034256
84.H	5.847377	-0.659067	0.403990
85.H	-2.526027	-4.547102	0.433051
86.H	-3.380819	2.745315	0.444619
87.H	1.685841	5.243844	0.537596
88.H	-0.020225	-4.206366	0.561647
89.H	-3.258766	1.019553	0.739667
90.H	-0.517045	-5.873978	0.919503
91.H	5.403478	2.924617	0.951664
92.H	-5.107379	-3.767366	1.200629
93.H	-6.122949	-2.323241	1.333839
94.H	1.396918	2.805733	1.025245
95.H	2.668362	4.767046	1.933287
96.H	-3.133090	4.328268	1.863174
97.H	-4.111485	-0.921797	1.860820
98.H	3.987655	2.857271	2.030432
99.H	-2.646635	-6.350333	2.149314
100.H	1.158621	5.658251	2.179548
101.H	5.362297	1.760061	2.292214
102.H	-0.045125	-4.786559	2.234611
103.H	-1.627872	6.272696	2.323061
104.H	-3.861999	-5.096637	2.456760
105.H	3.069814	-3.590416	2.218723
106.H	5.507451	-1.694072	2.371500
107.H	-1.375163	0.333545	2.552016
108.H	-0.070799	-1.821075	2.645803
109.H	5.404415	-0.139167	3.234498
110.H	-2.923025	2.160495	3.048443
111.H	-2.390776	-5.171373	3.446694
112.H	0.563397	1.979598	3.217551
113.H	1.576101	-3.231782	3.110984
114.H	2.083108	2.863691	3.383537
115.H	-5.569122	-1.903698	3.634797
116.H	-0.872991	5.384210	3.660778
117.H	-2.585497	5.829441	3.750570
118.H	-4.493295	-3.304006	3.779777
119.H	0.562334	3.643652	3.857274
120.H	3.081925	-3.544973	4.001782
121.H	-2.396677	-0.222231	3.880913
122.H	5.309787	-1.663454	4.137348
123.H	-1.646269	2.811569	4.087006
124.H	-3.911396	-1.689968	4.224859
125.H	-3.289572	3.471767	4.188784
126.H	-0.675919	0.075603	4.155765
127.H	-0.711259	-3.681999	4.181800

128.H	-2.008992	-2.696528	4.878031
129.H	2.720915	0.633506	4.712701
130.H	1.352051	-0.494624	4.707757
131.H	-0.317638	-2.217356	5.098943
132.H	2.858556	-0.937309	5.545909
133.N	0.368072	-0.026753	-2.413238
134.N	-2.411652	0.329556	-1.743008
135.N	-1.632461	-1.646475	0.221994
136.N	-1.357248	1.962216	0.421305
137.P	2.265676	-0.232407	1.214484
138.Si	1.412989	-1.066443	-3.425697
139.Si	3.948815	0.997286	0.330282
140.Si	-0.933826	3.579052	1.049326
141.Si	-2.212166	-2.473552	1.692735
142.Si	3.119034	-1.230696	3.071485
143.U	-0.200191	0.057390	-0.193707

Energy: −759.98555202 eV

Table S6. Final Coordinates and Single Point Energy of 4UAs After Geometry Optimisation

1.C	-0.100273	-0.440090	-6.058248
2.C	2.690131	-2.703401	-5.196101
3.C	0.835317	1.696513	-5.093850
4.C	1.004219	0.166445	-5.164989
5.C	0.689679	-3.610406	-3.967709
6.C	1.830003	-2.573703	-3.918884
7.C	4.178916	-0.087162	-3.294131
8.C	-2.289658	-0.002780	-2.957087
9.C	-1.314746	-1.169257	-2.844682
10.C	2.829106	0.010010	-2.557234
11.C	2.632824	1.442692	-2.038939
12.C	-2.644095	2.068739	-1.662618
13.C	-1.264986	2.709923	-1.549276
14.C	0.769245	5.483683	-1.173800
15.C	-3.627716	-0.060762	-0.887146
16.C	1.526182	-3.705088	-0.350683
17.C	4.502877	-2.886104	-0.253872
18.C	-2.609544	-4.121231	-0.174393
19.C	1.002473	4.909013	0.238687
20.C	2.426913	4.335221	0.347993
21.C	-4.858409	-3.402250	0.723629
22.C	-3.355134	-0.062190	0.612509
23.C	-2.009482	4.743403	0.972478
24.C	-3.376434	-3.648583	1.072469
25.C	5.033904	1.249876	1.284060
26.C	-1.809809	6.198175	1.448715
27.C	-3.124486	4.072924	1.799300
28.C	3.117511	-4.048645	2.225400
29.C	0.224354	2.963675	2.514053
30.C	-0.836639	-2.909072	2.730787
31.C	-0.877113	-4.418888	3.045966
32.C	5.101733	-1.118923	3.232769
33.C	-0.680862	1.892191	3.144082
34.C	-3.727998	-1.681742	3.413274
35.C	0.565016	4.043586	3.561461
36.C	3.382786	1.291704	3.880944

37.C	-0.303312	-2.132153	3.949994
38.C	-3.390397	-0.372781	4.150412
39.C	-3.997446	-2.822910	4.418839
40.H	-0.075114	0.014729	-7.064224
41.H	0.005096	-1.526847	-6.187282
42.H	2.095169	-2.515138	-6.103590
43.H	0.695032	2.118672	-6.104203
44.H	1.962372	-0.019997	-5.690174
45.H	-1.107033	-0.250016	-5.653255
46.H	3.096249	-3.725540	-5.284696
47.H	3.542352	-2.010893	-5.215309
48.H	0.013347	-3.434707	-4.819156
49.H	1.709475	2.192849	-4.652220
50.H	-0.044251	1.989591	-4.498337
51.H	4.176326	0.483705	-4.236757
52.H	1.099704	-4.627103	-4.093562
53.H	-1.225380	-1.653884	-3.828913
54.H	4.454213	-1.123386	-3.532501
55.H	-1.841840	0.764163	-3.602276
56.H	-3.257437	-0.300740	-3.403240
57.H	2.474729	-2.855572	-3.064934
58.H	0.084608	-3.613675	-3.051971
59.H	2.676224	2.185444	-2.848099
60.H	4.987575	0.327363	-2.668893
61.H	-3.175971	2.369792	-2.585530
62.H	-0.645160	2.391756	-2.406489
63.H	-1.756939	-1.942056	-2.187899
64.H	0.877462	4.705597	-1.944971
65.H	2.897844	-0.627035	-1.653819
66.H	-1.378563	3.799784	-1.663630
67.H	1.672864	1.604056	-1.518822
68.H	1.509447	6.269009	-1.403074
69.H	3.425060	1.693213	-1.318603
70.H	-0.228769	5.933093	-1.290392
71.H	-3.695762	-1.101725	-1.230087
72.H	-4.583582	0.440805	-1.133156
73.H	4.411898	-2.311311	-1.186716
74.H	1.925363	-4.579741	-0.888257
75.H	1.124319	-3.005777	-1.097448
76.H	-3.249203	2.390270	-0.805233
77.H	-2.623569	-3.351347	-0.961875
78.H	-3.061265	-5.031542	-0.603507
79.H	4.795264	-3.917146	-0.514476
80.H	2.550688	3.446536	-0.285428
81.H	3.175524	5.076699	0.020325
82.H	-2.377052	4.806239	-0.070127
83.H	-4.981740	-2.579167	0.002287
84.H	-5.305183	-4.298596	0.259555
85.H	-1.553990	-4.344178	0.035565
86.H	5.323826	-2.443243	0.328628
87.H	0.690424	-4.053874	0.272214
88.H	5.418807	0.651587	0.445235
89.H	-1.131956	6.764276	0.794522
90.H	0.936340	5.756239	0.948056
91.H	4.452518	2.083302	0.863924

92.H	-3.280879	0.983820	0.962676
93.H	-4.250127	-0.466870	1.112062
94.H	-2.773916	6.735083	1.465501
95.H	2.683992	4.047142	1.376897
96.H	-5.463723	-3.158978	1.608716
97.H	-4.084194	4.601929	1.670178
98.H	-3.284626	3.021084	1.520755
99.H	5.895473	1.676114	1.825385
100.H	-3.357056	-4.480629	1.801772
101.H	3.348013	-5.070940	1.879824
102.H	-1.098236	-5.032490	2.161325
103.H	-1.398522	6.242336	2.469215
104.H	-0.094019	-2.755976	1.924671
105.H	5.535753	-1.809943	2.497678
106.H	1.170481	2.459640	2.236730
107.H	-0.972776	1.087152	2.448379
108.H	3.938775	-3.717958	2.875782
109.H	2.201855	-4.094622	2.832615
110.H	-2.888935	4.092514	2.874204
111.H	-4.684110	-1.507070	2.884461
112.H	1.265848	4.799425	3.179209
113.H	0.095699	-4.755055	3.441307
114.H	5.935458	-0.603584	3.738364
115.H	2.793691	2.152061	3.534678
116.H	-1.632020	-4.658551	3.810593
117.H	-1.614319	2.326542	3.527933
118.H	-3.285708	0.473097	3.457984
119.H	-0.341012	4.571714	3.896988
120.H	-4.326809	-3.749335	3.925232
121.H	4.565588	-1.712185	3.987067
122.H	-0.212525	-1.057731	3.747120
123.H	-0.174610	1.403781	3.991271
124.H	0.704240	-2.489133	4.219478
125.H	1.024173	3.589980	4.455174
126.H	4.255810	1.683414	4.429486
127.H	2.764001	0.720342	4.588233
128.H	-2.450688	-0.450902	4.714927
129.H	-0.949346	-2.269022	4.830393
130.H	-3.100326	-3.064366	5.009729
131.H	-4.185076	-0.115659	4.871965
132.H	-4.786182	-2.533341	5.134549
133.As	2.093647	-0.747726	1.330622
134.N	-0.010258	-0.700060	-2.307921
135.N	-2.500559	0.588225	-1.606673
136.N	-0.629596	2.328911	-0.259378
137.N	-2.118083	-0.823386	0.947199
138.Si	1.329701	-0.747078	-3.481013
139.Si	2.871709	-2.905024	0.727479
140.Si	-0.374938	3.697532	0.848128
141.Si	-2.481908	-2.208913	2.019969
142.Si	3.969943	0.192633	2.449274
143.U	-0.303083	0.071070	-0.170170

Energy: −759.22758577 eV

Table S7. Final Coordinates and Single Point Energy of 4USb After Geometry Optimisation

1.C	-3.244307	-2.336105	-5.081214
2.C	-3.448843	1.298830	-5.032095
3.C	-0.228858	-0.418812	-5.137406
4.C	3.849362	-3.172826	-3.745576
5.C	-3.586801	0.845029	-3.560768
6.C	-2.690056	-2.278518	-3.640809
7.C	-0.536630	-0.024652	-3.679084
8.C	-0.151121	1.446296	-3.437009
9.C	-5.068859	0.508975	-3.282043
10.C	0.975916	-3.630500	-2.956826
11.C	-3.564337	-3.144662	-2.712593
12.C	2.290309	4.017997	-1.795147
13.C	6.165025	-1.545515	-1.505033
14.C	0.288358	5.531119	-1.473752
15.C	3.232920	-4.507400	-1.040568
16.C	1.439327	4.785992	-0.766705
17.C	5.521368	1.272315	-0.555496
18.C	-3.330200	-0.705654	-0.473985
19.C	-1.622632	2.996072	-0.055794
20.C	-3.788412	0.593121	0.176719
21.C	-0.855810	-3.621094	0.414901
22.C	-2.623101	2.591418	1.021288
23.C	5.351775	-1.104465	1.396171
24.C	2.466531	2.812434	1.385868
25.C	0.121960	-3.461131	1.592393
26.C	3.578333	3.797569	1.800363
27.C	0.772199	6.300840	2.129172
28.C	0.093434	4.916356	2.032267
29.C	0.469497	-4.843273	2.183187
30.C	-2.825526	0.591319	2.451834
31.C	2.230002	1.782057	2.501739
32.C	-3.141335	-3.294762	3.096140
33.C	-1.425024	0.350902	3.004042
34.C	-0.070011	4.325907	3.446811
35.C	2.416958	-1.953178	3.522429
36.C	-1.977235	-2.754729	3.950638
37.C	1.043526	-2.073906	4.204967
38.C	-1.676427	-3.742702	5.099695
39.C	0.857034	-0.955888	5.249904
40.H	-2.605565	-1.811601	-5.806177
41.H	-3.695529	0.487940	-5.733797
42.H	-0.858090	0.126271	-5.856810
43.H	-3.330296	-3.384334	-5.417618
44.H	0.821323	-0.185298	-5.380267
45.H	-2.437356	1.650837	-5.274975
46.H	-4.144213	2.130261	-5.241123
47.H	-4.251659	-1.894375	-5.146821
48.H	-0.375202	-1.492945	-5.318324
49.H	3.711538	-4.123970	-4.288636
50.H	3.632506	-2.349630	-4.442637
51.H	-0.763245	2.137659	-4.034568
52.H	-5.400842	-0.365338	-3.862462
53.H	0.714250	-2.976788	-3.801283
54.H	-1.691918	-2.755863	-3.657164

55.H	0.903101	1.616038	-3.704230
56.H	-5.715824	1.353380	-3.576581
57.H	4.906189	-3.103298	-3.454789
58.H	-3.624306	-4.175781	-3.099077
59.H	1.015994	-4.664666	-3.337080
60.H	-3.324806	1.721548	-2.934745
61.H	0.149272	-0.633581	-3.052503
62.H	6.066142	-1.258399	-2.561019
63.H	-4.597636	-2.768621	-2.649947
64.H	2.574369	4.674024	-2.635923
65.H	-0.256923	1.762272	-2.384216
66.H	-5.277861	0.296986	-2.224834
67.H	0.166635	-3.573463	-2.218548
68.H	1.742989	3.162888	-2.215359
69.H	0.684442	6.243207	-2.216841
70.H	-0.367419	4.834126	-2.017201
71.H	-3.161097	-3.198797	-1.693239
72.H	5.996778	-2.628438	-1.425468
73.H	5.384993	1.630079	-1.586512
74.H	7.204441	-1.346479	-1.194530
75.H	3.168499	-5.499514	-1.519970
76.H	3.219579	3.626134	-1.359379
77.H	-4.150181	-1.096143	-1.095976
78.H	4.273817	-4.355900	-0.717353
79.H	-0.339475	6.105334	-0.775233
80.H	-1.980248	2.623662	-1.034472
81.H	-3.964496	1.334019	-0.614193
82.H	2.089702	5.557649	-0.311147
83.H	6.588105	1.375097	-0.294657
84.H	-0.455881	-4.333231	-0.323562
85.H	2.602951	-4.520001	-0.138474
86.H	-1.624348	4.093245	-0.152159
87.H	-1.048097	-2.680304	-0.128733
88.H	4.949379	1.933111	0.110526
89.H	-3.172547	-1.466944	0.309860
90.H	2.829611	2.253654	0.502394
91.H	-4.734607	0.468565	0.738086
92.H	-1.833241	-4.005312	0.736750
93.H	-3.621744	3.038951	0.856782
94.H	3.831448	4.508799	1.001497
95.H	0.794624	6.829518	1.166109
96.H	1.055323	-3.030530	1.179155
97.H	6.422578	-0.968274	1.625187
98.H	5.105201	-2.166935	1.537570
99.H	0.903075	-5.500699	1.411232
100.H	-0.926415	5.099855	1.641381
101.H	4.502392	3.257332	2.065256
102.H	-2.252618	2.947334	1.990945
103.H	4.771035	-0.529501	2.131233
104.H	-0.425248	-5.353146	2.573705
105.H	1.481703	1.017347	2.236627
106.H	1.810161	6.223939	2.488560
107.H	3.285200	4.383109	2.685610
108.H	0.231249	6.944249	2.844343
109.H	-2.901331	-4.281760	2.672677

110.H	-3.355921	-0.369181	2.422525
111.H	-3.396041	-2.632386	2.255622
112.H	3.160099	1.242775	2.738075
113.H	2.664546	-2.845034	2.930034
114.H	1.197227	-4.777732	3.004671
115.H	-3.414432	1.285591	3.081815
116.H	2.458166	-1.094099	2.838329
117.H	-0.877584	1.308713	3.029963
118.H	1.885589	2.255814	3.431343
119.H	-0.534370	3.329284	3.442030
120.H	-4.051410	-3.420250	3.707470
121.H	0.902735	4.228134	3.952490
122.H	-0.695314	4.980631	4.077453
123.H	-1.506534	0.032638	4.055699
124.H	3.218605	-1.821022	4.268708
125.H	-2.339541	-1.826489	4.432167
126.H	-1.274001	-4.697378	4.726650
127.H	1.029197	-3.036803	4.749601
128.H	0.871438	0.037871	4.777936
129.H	-2.599277	-3.977305	5.657544
130.H	1.673796	-0.973262	5.991162
131.H	-0.952509	-3.339076	5.821289
132.H	-0.089005	-1.048459	5.805201
133.N	-2.077509	-0.477550	-1.238190
134.N	-0.278777	2.449487	0.270121
135.N	-2.714274	1.108269	1.062919
136.N	-0.717445	-0.633508	2.147758
137.Sb	2.500137	-0.823138	-1.085005
138.Si	-2.277280	-0.492218	-3.013755
139.Si	2.672230	-3.147906	-2.250330
140.Si	4.998531	-0.545674	-0.385434
141.Si	0.897808	3.703226	0.737330
142.Si	-0.406498	-2.201927	2.941028
143.U	-0.331502	0.190551	0.071303

Energy: −758.12643911 eV

Table S8. Final Coordinates and Single Point Energy of 3ThP After Geometry Optimisation

1.C	0.203152	-0.610879	-3.760435
2.C	3.514651	1.514320	-3.287191
3.C	1.492316	-1.084892	-3.094325
4.C	-2.032947	0.221280	-3.112571
5.C	-1.516757	5.191439	-2.754661
6.C	4.873890	-1.709904	-2.640999
7.C	-1.665076	1.659813	-2.733962
8.C	-1.392488	-2.132084	-2.706349
9.C	0.812384	4.491973	-2.116012
10.C	6.064993	0.146557	-1.442212
11.C	-0.589511	4.831134	-1.570763
12.C	4.767120	-0.691814	-1.483996
13.C	-2.379018	-2.307575	-1.548116
14.C	-0.482810	6.065502	-0.645661
15.C	-4.781147	-0.325375	-0.162728
16.C	-3.118079	3.689263	-0.160172
17.C	4.625157	-1.465355	-0.160010
18.C	3.146207	1.687834	-0.259192

19.C	-5.194247	-3.354101	0.866805
20.C	1.028943	-3.481618	0.769946
21.C	-0.329085	3.159159	1.061876
22.C	-2.891034	-3.827837	1.739446
23.C	-3.993478	-2.755092	1.635286
24.C	-2.682817	0.112660	1.970169
25.C	3.283681	-3.253354	2.838399
26.C	-4.461092	-2.384036	3.061182
27.C	0.448914	-3.564451	3.821946
28.C	1.504916	2.136186	4.338909
29.C	-0.464542	-0.000629	5.308312
30.C	2.540293	-0.524630	5.450709
31.H	-0.012010	-1.170475	-4.692999
32.H	3.724423	0.913551	-4.185176
33.H	-2.312713	0.165250	-4.182974
34.H	0.318451	0.450153	-4.018589
35.H	2.312280	-0.910550	-3.811630
36.H	4.968129	-1.220451	-3.623045
37.H	-1.119628	6.072719	-3.289664
38.H	-1.596157	4.375495	-3.487532
39.H	2.635971	2.142562	-3.498366
40.H	-1.839207	-2.475445	-3.660257
41.H	-0.947294	2.058201	-3.476129
42.H	4.372628	2.190275	-3.144453
43.H	1.465771	-2.181485	-2.956675
44.H	1.214784	5.337276	-2.702443
45.H	5.769315	-2.343306	-2.506056
46.H	0.795168	3.608467	-2.770525
47.H	-2.589093	2.252892	-2.871407
48.H	-2.534512	5.443489	-2.420377
49.H	4.003859	-2.382117	-2.676835
50.H	6.234552	0.699356	-2.379260
51.H	-2.899851	-0.085056	-2.516118
52.H	-0.500642	-2.751383	-2.526059
53.H	-3.397266	-2.061902	-1.906131
54.H	6.936615	-0.515499	-1.290574
55.H	-0.094952	6.929925	-1.214105
56.H	1.530808	4.289542	-1.308160
57.H	-2.407327	-3.387085	-1.307136
58.H	-3.760439	3.610672	-1.050541
59.H	-5.213820	-0.929126	-0.974152
60.H	6.062849	0.874228	-0.616027
61.H	-4.457276	0.637290	-0.586546
62.H	-1.460008	6.361322	-0.232551
63.H	2.380154	2.457081	-0.445468
64.H	4.103293	2.220922	-0.149411
65.H	5.493633	-2.131371	-0.006880
66.H	-4.933758	-3.632299	-0.166631
67.H	3.721009	-2.090017	-0.157682
68.H	0.202564	5.894758	0.198435
69.H	-3.252058	4.701562	0.253065
70.H	1.625850	-3.019898	-0.034653
71.H	-5.595199	-0.115016	0.548806
72.H	-3.501296	2.974081	0.583353
73.H	4.560645	-0.794850	0.709752

74.H	-6.045358	-2.658501	0.821749
75.H	-0.039125	-3.329744	0.545333
76.H	-2.575090	-4.188766	0.749827
77.H	1.227422	-4.564770	0.738644
78.H	2.924971	1.192634	0.700800
79.H	0.757756	3.272484	0.933419
80.H	-5.544933	-4.271387	1.374062
81.H	-2.483996	1.073178	1.464295
82.H	-0.497000	2.205768	1.596630
83.H	-0.652051	3.939558	1.768488
84.H	-3.260044	-4.700066	2.308396
85.H	3.985812	-2.887565	2.077202
86.H	-1.996470	-3.451143	2.252109
87.H	-1.770027	-0.200466	2.501783
88.H	-3.455049	0.326815	2.723941
89.H	3.356644	-4.353813	2.873255
90.H	-5.232307	-1.597029	3.058775
91.H	-4.899375	-3.268425	3.558305
92.H	3.612485	-2.863610	3.812996
93.H	-3.627232	-2.034116	3.688332
94.H	0.525407	-4.661800	3.748870
95.H	-0.613572	-3.292790	3.766576
96.H	0.745790	2.668974	3.748247
97.H	2.489020	2.356982	3.900059
98.H	0.813041	-3.274867	4.818892
99.H	-1.297797	0.514052	4.806913
100.H	3.535954	-0.370238	5.010436
101.H	1.486296	2.548846	5.361024
102.H	-0.720959	-1.068394	5.374950
103.H	2.390145	-1.606663	5.576716
104.H	-0.392358	0.394930	6.335335
105.H	2.535202	-0.070620	6.455561
106.N	-0.929544	-0.726675	-2.808653
107.N	1.693805	-0.385088	-1.806733
108.N	-1.125731	1.776652	-1.365410
109.N	-1.994736	-1.486799	-0.389386
110.P	1.194160	-0.514500	2.270482
111.Si	3.237927	0.475557	-1.710909
112.Si	-1.296191	3.328538	-0.568380
113.Si	-3.323368	-1.168627	0.730151
114.Si	1.494358	-2.736768	2.463671
115.Si	1.178263	0.266429	4.385650
116.Th	-0.125446	-0.078710	-0.366425

Energy: –616.01263751 eV

Table S9. Final Coordinates and Single Point Energy of 3ThAs After Geometry Optimisation

1.C	-0.325059	-0.982179	-6.060972
2.C	-2.714977	-0.520184	-5.456470
3.C	-1.252098	-0.390867	-4.973478
4.C	-0.916919	1.102770	-4.792446
5.C	-1.621319	-3.130165	-3.570292
6.C	0.878231	-1.410030	-2.933585
7.C	4.548414	-1.234649	-2.260227
8.C	-3.160256	-0.408188	-1.918676
9.C	4.628645	1.727123	-1.494570

10.C	-1.589986	4.426217	-1.602389
11.C	0.912900	2.651305	-1.668099
12.C	-3.798207	-0.621633	-0.545974
13.C	1.231952	5.869152	-0.213949
14.C	6.090433	-0.338688	0.212249
15.C	-0.320975	-3.819094	0.196389
16.C	-3.508827	1.636816	0.411647
17.C	0.569760	4.757802	0.630909
18.C	-2.396192	2.493762	1.013770
19.C	1.659700	4.015954	1.426266
20.C	-0.420202	5.403117	1.625858
21.C	-2.957307	-3.917570	1.793301
22.C	-3.386699	-0.343073	1.882406
23.C	-2.046129	-0.669538	2.555626
24.C	4.214605	1.742822	2.870268
25.C	1.264540	-3.680120	3.128902
26.C	-0.488223	-5.455841	3.409067
27.C	-0.246124	-3.933031	3.306093
28.C	4.209150	-1.218472	3.517604
29.C	1.581698	0.393799	3.690264
30.C	-0.718943	-3.268121	4.617334
31.H	-0.489651	-0.462026	-7.021771
32.H	-2.839733	-0.004818	-6.425880
33.H	-0.515890	-2.052594	-6.235119
34.H	0.739371	-0.867754	-5.805544
35.H	-3.011850	-1.569492	-5.604667
36.H	-1.085161	1.654642	-5.734563
37.H	-3.427445	-0.064917	-4.752931
38.H	-1.282957	-3.544730	-4.532896
39.H	0.136878	1.255472	-4.514706
40.H	-1.540579	1.570448	-4.016566
41.H	1.407570	-2.001811	-3.695925
42.H	-2.721251	-3.177620	-3.563004
43.H	-1.257324	-3.800382	-2.777571
44.H	5.465845	-1.063369	-2.847633
45.H	1.341402	-0.410347	-2.934262
46.H	3.689013	-1.101391	-2.931852
47.H	-3.670492	-1.114609	-2.599720
48.H	-1.073211	5.172159	-2.226727
49.H	5.576693	1.844147	-2.046494
50.H	0.392550	2.065174	-2.443134
51.H	-3.420203	0.598296	-2.296995
52.H	3.805850	1.945210	-2.191035
53.H	-2.109249	3.732891	-2.281802
54.H	1.524127	3.395685	-2.200265
55.H	1.125703	-1.891715	-1.969994
56.H	4.550239	-2.280677	-1.920076
57.H	-2.354664	4.964924	-1.022480
58.H	1.618923	1.993107	-1.128182
59.H	1.963504	5.464376	-0.930579
60.H	4.604475	2.485582	-0.698174
61.H	0.493786	6.458161	-0.779915
62.H	6.959519	-0.236683	-0.459092
63.H	-4.888518	-0.425555	-0.590206
64.H	-3.600761	1.895167	-0.652138

65.H	-0.894569	-3.523882	-0.694863
66.H	-3.654209	-1.671557	-0.262386
67.H	1.776522	6.570901	0.443264
68.H	-0.234741	-4.915949	0.177656
69.H	0.698387	-3.408567	0.109889
70.H	6.113875	-1.347834	0.647701
71.H	6.214905	0.388602	1.026444
72.H	-4.486762	1.829813	0.896802
73.H	2.405033	3.551965	0.763928
74.H	-2.687802	3.551881	0.898694
75.H	-1.221969	5.959950	1.116053
76.H	-3.521316	-3.610555	0.898980
77.H	-2.941268	-5.018511	1.804194
78.H	2.194772	4.713366	2.095194
79.H	-3.989586	-1.257524	1.802619
80.H	0.110011	6.119957	2.278859
81.H	-2.346742	2.323350	2.105959
82.H	1.233351	3.216764	2.048762
83.H	3.796403	2.586512	2.306077
84.H	-0.887819	4.652677	2.280584
85.H	1.678329	-4.248773	2.283504
86.H	-0.213138	-5.985008	2.482979
87.H	-3.961921	0.381941	2.483352
88.H	5.253357	1.592616	2.541840
89.H	-3.528218	-3.594004	2.678182
90.H	-1.549147	0.288355	2.822137
91.H	5.170426	-1.350947	2.999165
92.H	1.483694	-2.620276	2.942359
93.H	3.689041	-2.185903	3.510551
94.H	1.054838	1.308382	3.376344
95.H	-1.538040	-5.695529	3.636141
96.H	-2.234545	-1.154198	3.527990
97.H	0.894055	-0.455339	3.563241
98.H	4.245314	2.028857	3.935884
99.H	1.815830	-3.986727	4.035433
100.H	0.128846	-5.880508	4.221123
101.H	4.425238	-0.950113	4.565946
102.H	-0.485206	-2.193569	4.638164
103.H	1.806248	0.491503	4.764839
104.H	-1.802288	-3.387809	4.779759
105.H	-0.208647	-3.728597	5.482524
106.As	2.538455	-0.454344	0.482861
107.N	-1.698496	-0.610230	-1.895921
108.N	-1.109239	2.178078	0.363418
109.N	-3.150474	0.199815	0.508691
110.N	-1.179757	-1.465347	1.666841
111.Si	-0.979834	-1.357495	-3.313074
112.Si	4.499733	-0.036139	-0.786010
113.Si	-0.335779	3.492436	-0.514876
114.Si	-1.182245	-3.220004	1.771365
115.Si	3.183339	0.150954	2.686024
116.Th	-0.466565	-0.027778	0.007073

Energy: −615.23023502 eV

Table S10. Final Coordinates and Single Point Energy of 4ThP After Geometry Optimisation

1.C	1.114501	1.757731	-4.950730
2.C	1.880746	4.125949	-4.552819
3.C	-1.762290	4.218256	-3.993999
4.C	-0.001145	-2.864184	-3.931035
5.C	1.528070	2.782848	-3.876110
6.C	-1.892945	-4.471584	-3.471415
7.C	-0.477990	-4.029200	-3.046289
8.C	-1.402433	0.906700	-2.913165
9.C	-0.898954	4.384729	-2.724330
10.C	3.044200	-0.411212	-2.551367
11.C	-2.798356	0.921293	-2.300131
12.C	2.531938	-4.424128	-1.831660
13.C	-1.784958	4.839404	-1.548727
14.C	-3.155073	-1.364047	-1.458174
15.C	5.027399	1.472507	-1.162872
16.C	2.397200	4.590106	-1.056925
17.C	-0.365796	-6.571583	-0.663738
18.C	5.293944	-1.556695	-0.859814
19.C	1.676200	-3.615640	-0.834650
20.C	1.465117	3.380297	-0.851202
21.C	-1.040092	-5.237468	-0.275385
22.C	-2.450687	-2.319619	-0.501894
23.C	-3.721031	0.571594	-0.044432
24.C	0.752161	3.514452	0.505188
25.C	2.081074	-3.967495	0.610154
26.C	-2.934706	1.516046	0.858797
27.C	-1.194669	-5.125569	1.253420
28.C	5.672139	0.020960	2.376117
29.C	3.715387	2.277511	3.009945
30.C	-0.467038	-1.868175	3.206580
31.C	-3.948780	-1.441588	3.497795
32.C	-2.684415	3.476881	3.695591
33.C	-3.708656	0.057257	3.766192
34.C	-0.510907	-0.465696	3.840720
35.C	-0.255286	2.916620	4.093513
36.C	3.268647	-0.545586	4.104886
37.C	-1.697255	2.381581	4.150090
38.C	-4.130024	0.401711	5.211132
39.C	-0.417919	-0.588286	5.375879
40.H	1.911459	1.647085	-5.705693
41.H	0.207889	2.073317	-5.490752
42.H	2.690559	3.986349	-5.289258
43.H	1.019336	4.542216	-5.098073
44.H	0.048460	-3.159717	-4.993446
45.H	-1.178211	3.899404	-4.869172
46.H	-1.920926	-4.710884	-4.548464
47.H	0.923447	0.762780	-4.527580
48.H	-2.252306	5.171774	-4.255900
49.H	2.216578	4.891378	-3.839888
50.H	-0.695058	-2.011292	-3.866464
51.H	-2.567275	3.481416	-3.846153
52.H	-1.418758	1.510457	-3.833332
53.H	2.459447	2.398433	-3.418293
54.H	0.993080	-2.490395	-3.646799

55.H	0.194470	-4.885022	-3.246474
56.H	-2.639187	-3.678277	-3.304573
57.H	3.655433	-0.139206	-3.425960
58.H	-1.170501	-0.119526	-3.251404
59.H	-0.197997	5.217352	-2.933350
60.H	-3.576042	0.614628	-3.026271
61.H	-2.235226	-5.365920	-2.930765
62.H	2.384069	-4.109541	-2.874224
63.H	2.765806	-1.468313	-2.663901
64.H	2.125549	0.192033	-2.594325
65.H	-2.788338	-1.555821	-2.475371
66.H	5.717581	1.373907	-2.017349
67.H	-3.025195	1.945875	-1.977436
68.H	-2.436457	5.677364	-1.852216
69.H	2.309818	-5.501103	-1.778773
70.H	-0.324186	-6.717784	-1.753512
71.H	3.067049	4.462600	-1.917839
72.H	5.841892	-1.629629	-1.814896
73.H	3.604034	-4.305999	-1.603788
74.H	-4.251870	-1.518423	-1.458611
75.H	4.371889	2.334521	-1.352702
76.H	1.830274	5.522589	-1.208890
77.H	-2.444948	4.031233	-1.194488
78.H	1.934334	-2.547110	-0.968298
79.H	-1.195315	5.179657	-0.687543
80.H	-2.063956	-5.280041	-0.693118
81.H	4.790654	-2.518152	-0.682808
82.H	-2.803711	-3.338750	-0.727061
83.H	-0.918516	-7.427237	-0.239214
84.H	2.110889	2.483313	-0.764190
85.H	0.665177	-6.632864	-0.282642
86.H	-4.604252	1.064145	-0.494617
87.H	5.630573	1.705816	-0.273492
88.H	3.034294	4.740265	-0.169524
89.H	6.032012	-1.408715	-0.059435
90.H	-2.574827	2.372230	0.260988
91.H	-4.079195	-0.270322	0.562231
92.H	-2.804258	-2.115996	0.525958
93.H	0.259317	4.491513	0.612113
94.H	-0.038169	2.762756	0.683009
95.H	3.146316	-3.739148	0.777128
96.H	1.935431	-5.038898	0.817187
97.H	1.513151	-3.392022	1.352876
98.H	1.477998	3.419478	1.325867
99.H	-1.780323	-5.972801	1.649437
100.H	6.148309	0.535890	1.531117
101.H	-1.706824	-4.200183	1.549853
102.H	-3.633478	1.950598	1.591683
103.H	-0.220826	-5.137517	1.762993
104.H	5.832587	-1.059698	2.254492
105.H	4.068693	2.903560	2.177208
106.H	-0.570100	-1.878689	2.106697
107.H	-3.633025	-1.748235	2.489815
108.H	-2.485389	3.789237	2.659189
109.H	6.194550	0.337107	3.294141

110.H	-4.394949	0.616257	3.100772
111.H	0.053635	3.125642	3.060080
112.H	2.682468	2.576586	3.239782
113.H	-5.017219	-1.694912	3.604050
114.H	0.485343	-2.369916	3.436772
115.H	-3.734590	3.152439	3.750288
116.H	4.336470	2.503011	3.893109
117.H	0.396931	0.070469	3.499985
118.H	-1.276085	-2.506491	3.586965
119.H	3.323153	-1.629108	3.925184
120.H	-2.587873	4.376628	4.326405
121.H	-3.398012	-2.066690	4.217205
122.H	2.241187	-0.303082	4.407887
123.H	0.471001	2.210759	4.521096
124.H	-0.162990	3.860247	4.657954
125.H	3.930431	-0.306235	4.953954
126.H	-1.923196	2.161206	5.211030
127.H	-4.118186	1.483171	5.405993
128.H	-5.154758	0.043745	5.410396
129.H	0.478877	-1.157185	5.671615
130.H	-1.288119	-1.124574	5.784478
131.H	-3.472319	-0.075291	5.954570
132.H	-0.365618	0.390131	5.874058
133.N	-0.395024	1.380780	-1.927918
134.N	-2.826710	0.039393	-1.104626
135.N	-0.967838	-2.194826	-0.587493
136.N	-1.803034	0.796402	1.499736
137.P	2.568545	-0.111458	0.768435
138.Si	0.344976	2.944113	-2.345146
139.Si	-0.218197	-3.714120	-1.150268
140.Si	4.034161	-0.136728	-0.950863
141.Si	3.826233	0.431166	2.576698
142.Si	-1.937913	0.680628	3.266177
143.Th	-0.266940	-0.011323	-0.058102

Energy: −760.27565310 eV

Table S11. Final Coordinates and Single Point Energy of 4ThAs After Geometry Optimisation

1.C	-0.484392	2.326179	-6.053062
2.C	-2.467467	-0.772479	-5.614424
3.C	1.111173	-0.855720	-5.000444
4.C	-3.705494	1.121117	-4.516934
5.C	-0.403468	2.350021	-4.512323
6.C	-2.605615	0.054668	-4.317334
7.C	0.979607	2.850463	-4.053111
8.C	0.424777	-0.632199	-3.638132
9.C	4.340735	0.179309	-3.063600
10.C	-0.075493	-1.975496	-3.072179
11.C	4.006094	-2.818349	-2.447618
12.C	-2.026137	-4.697155	-1.455049
13.C	-2.103985	2.148214	-1.536400
14.C	6.075841	-1.142891	-0.937807
15.C	-3.325230	1.522437	-0.863892
16.C	1.562379	3.449223	-0.368203
17.C	-2.301340	-4.764000	0.058826
18.C	-3.016287	-1.570874	-0.079068

19.C	1.166066	-4.239288	0.069965
20.C	-2.070743	-6.192638	0.596365
21.C	-3.666876	-0.397802	0.649118
22.C	1.928194	3.154830	1.096487
23.C	0.468844	-3.738961	1.348625
24.C	3.121396	4.028422	1.529953
25.C	-2.942175	1.773227	1.559486
26.C	0.102541	5.999484	1.874159
27.C	-0.697308	4.687930	2.041671
28.C	4.424497	-2.599664	2.262519
29.C	4.976320	0.415274	2.409327
30.C	0.820492	-4.666233	2.530518
31.C	-1.777160	1.454085	2.492462
32.C	-3.767635	-3.650407	2.908563
33.C	-2.228324	-3.550162	2.885205
34.C	-1.803462	4.912471	3.097026
35.C	2.262404	-0.716730	3.321977
36.C	-1.748847	-2.460965	3.859065
37.C	1.111983	2.930686	4.079938
38.C	1.562724	4.258701	4.726931
39.C	0.237615	2.141712	5.074208
40.H	-0.217511	3.314565	-6.465609
41.H	0.213960	1.596805	-6.491521
42.H	-1.491370	2.081449	-6.419939
43.H	-2.089058	-0.165369	-6.450560
44.H	-3.450085	-1.169411	-5.922583
45.H	0.402155	-1.213230	-5.761438
46.H	-1.792235	-1.630590	-5.498097
47.H	-3.415177	1.863089	-5.276162
48.H	1.575744	0.060941	-5.388423
49.H	1.905945	-1.614530	-4.914217
50.H	-4.639604	0.650823	-4.868403
51.H	1.195013	3.849332	-4.468251
52.H	1.784846	2.180879	-4.392180
53.H	-1.144886	3.098228	-4.169753
54.H	5.088474	-0.096873	-3.826013
55.H	-3.947672	1.673266	-3.599065
56.H	-2.960452	-0.640792	-3.531288
57.H	-0.829891	-2.433311	-3.727932
58.H	3.366655	0.262420	-3.564982
59.H	4.785510	-3.056214	-3.191522
60.H	1.049256	2.920273	-2.961179
61.H	3.031881	-2.851860	-2.957021
62.H	1.205350	-0.252326	-2.946745
63.H	0.750379	-2.696488	-2.974987
64.H	4.602913	1.172571	-2.670816
65.H	-2.435595	2.714754	-2.422557
66.H	-2.631960	-5.444838	-1.995574
67.H	-0.546450	-1.899955	-2.076121
68.H	4.007678	-3.609335	-1.683398
69.H	6.788713	-1.405146	-1.737189
70.H	-2.268898	-3.710457	-1.872437
71.H	-0.971519	-4.901497	-1.689255
72.H	-3.740726	0.760393	-1.536121
73.H	-3.040620	-1.369814	-1.167265

74.H	2.431199	3.284484	-1.024209
75.H	-1.676365	2.908400	-0.856382
76.H	6.367136	-0.161620	-0.537016
77.H	-4.120040	2.265798	-0.662416
78.H	0.991727	-3.574929	-0.785791
79.H	0.752060	2.806411	-0.753303
80.H	1.229060	4.487912	-0.511810
81.H	6.187290	-1.887230	-0.137773
82.H	-2.682586	-6.923451	0.039917
83.H	0.830741	-5.251512	-0.201420
84.H	-3.379852	-4.555949	0.191091
85.H	-3.664654	-2.451118	0.063130
86.H	2.256956	-4.281034	0.219841
87.H	-1.019793	-6.503482	0.487093
88.H	-4.727854	-0.272302	0.357605
89.H	3.963634	3.892505	0.831022
90.H	0.834085	5.946645	1.056938
91.H	2.292560	2.106599	1.112779
92.H	-1.199698	4.500165	1.072061
93.H	-2.333918	-6.288305	1.660528
94.H	-2.822922	2.800422	1.190671
95.H	2.875525	5.100778	1.535802
96.H	-0.580003	6.837702	1.650693
97.H	5.828355	0.329134	1.719348
98.H	0.911359	-2.744921	1.567191
99.H	-3.637095	-0.600192	1.727849
100.H	5.314881	-2.745408	1.634589
101.H	3.708551	-3.399052	2.023747
102.H	-3.920575	1.710124	2.073674
103.H	-4.140708	-4.491361	2.306032
104.H	4.579335	1.437031	2.322147
105.H	-4.247449	-2.733873	2.530390
106.H	3.486643	3.766792	2.532628
107.H	0.388684	-5.670169	2.401343
108.H	0.648310	6.264744	2.792132
109.H	1.912828	-4.794016	2.606658
110.H	-2.402416	5.804291	2.843223
111.H	-1.886328	0.413235	2.850089
112.H	1.614625	0.153529	3.142287
113.H	-2.502402	4.069786	3.184449
114.H	5.357895	0.279676	3.435599
115.H	4.735084	-2.722144	3.314118
116.H	-1.843335	-4.516963	3.263059
117.H	1.626740	-1.614290	3.336463
118.H	0.469956	-4.278248	3.496817
119.H	-1.868118	2.069061	3.400568
120.H	-2.151218	-1.474998	3.577646
121.H	-4.135250	-3.799362	3.938364
122.H	-0.653521	-2.364824	3.884743
123.H	-1.377294	5.088230	4.096537
124.H	2.229367	4.846764	4.081115
125.H	2.024459	2.324183	3.918011
126.H	2.697325	-0.605891	4.328251
127.H	-2.086324	-2.664728	4.889902
128.H	0.701010	4.895647	4.980042

129.H	-0.035459	1.149366	4.691217
130.H	-0.691817	2.680237	5.319003
131.H	2.105227	4.064216	5.668163
132.H	0.777571	1.990823	6.024791
133.As	2.647480	-0.545648	-0.096874
134.N	-1.102226	1.104793	-1.864849
135.N	-2.905928	0.852151	0.393836
136.N	-1.622104	-1.804662	0.389342
137.N	-0.478464	1.639468	1.795006
138.Si	-0.929693	0.732808	-3.595634
139.Si	4.322454	-1.112747	-1.672956
140.Si	-1.415826	-3.412066	1.129629
141.Si	3.642314	-0.882054	2.021211
142.Si	0.417298	3.104515	2.277707
143.Th	-0.274729	0.097984	0.055993

Energy: −759.46662399 eV

Table S12. Final Coordinates and Single Point Energy of 4ThSb After Geometry Optimisation

1.C	-0.311199	-0.434612	-6.566356
2.C	3.033805	0.572645	-5.534638
3.C	-2.693855	0.450098	-4.903333
4.C	-1.778312	-2.486209	-4.859488
5.C	1.290656	2.928932	-4.626746
6.C	3.504927	1.787631	-2.804422
7.C	-3.225552	2.760808	-2.062832
8.C	-3.944356	-3.403682	-1.702215
9.C	-0.417929	5.130860	-1.488694
10.C	4.661315	-1.699369	-1.180529
11.C	-0.499938	-4.893009	-1.094982
12.C	-2.897032	-2.475103	-1.053157
13.C	-3.456160	-1.044826	-0.961501
14.C	-0.252778	3.731203	-0.859384
15.C	-3.332685	3.435016	-0.683969
16.C	3.383351	-1.392294	-0.376093
17.C	1.166004	3.580007	-0.280811
18.C	2.558806	-2.679480	-0.196134
19.C	-4.569054	2.910593	0.073564
20.C	-1.421776	-4.923461	0.138110
21.C	6.348338	0.576112	1.014465
22.C	4.877637	1.031326	0.898896
23.C	-0.710346	-5.619822	1.317159
24.C	-2.649320	5.821518	1.520430
25.C	-4.306634	-4.839999	1.793279
26.C	4.677321	2.344518	1.681272
27.C	-1.856875	4.533085	1.832448
28.C	-3.518082	-3.521369	1.949879
29.C	-4.503126	-2.356125	2.170614
30.C	-2.291949	1.276886	2.094795
31.C	5.170223	-2.556540	2.378319
32.C	-0.518490	4.897151	2.506658
33.C	-0.455883	-2.360015	2.525076
34.C	4.060866	-1.534423	2.714878
35.C	1.754015	1.097885	2.719760
36.C	-1.424930	1.108817	3.340286
37.C	-0.945383	-1.306625	3.514845

38.C	0.889559	0.322912	3.710448
39.C	4.408400	-0.837658	4.048879
40.H	-1.033533	-0.663627	-7.367828
41.H	0.029311	0.599692	-6.713019
42.H	0.552837	-1.102266	-6.688102
43.H	2.355480	0.363696	-6.372394
44.H	3.799145	1.283836	-5.890778
45.H	-3.317633	0.220613	-5.784186
46.H	-2.475687	-2.661504	-5.695912
47.H	3.539287	-0.366329	-5.265483
48.H	0.504089	2.724795	-5.368141
49.H	-0.936127	-3.186450	-4.961375
50.H	2.029969	3.601702	-5.093910
51.H	-2.403653	1.509517	-4.958188
52.H	-3.316502	0.324356	-4.006366
53.H	-2.305900	-2.730225	-3.926774
54.H	0.822593	3.463553	-3.786904
55.H	4.148020	2.571296	-3.238028
56.H	-4.258298	-3.017449	-2.685651
57.H	4.139818	0.914582	-2.593723
58.H	-4.189749	2.797952	-2.597572
59.H	-2.475960	3.243740	-2.705135
60.H	0.346516	5.305606	-2.263786
61.H	4.397188	-2.160478	-2.146587
62.H	-2.938284	1.702949	-1.976639
63.H	-1.017673	-4.544189	-1.999357
64.H	-1.400804	5.265156	-1.962195
65.H	3.113986	2.162343	-1.849389
66.H	-3.562453	-4.422579	-1.856949
67.H	-3.727961	-0.667796	-1.959054
68.H	-2.022852	-2.438947	-1.732864
69.H	-0.348036	2.986625	-1.674947
70.H	5.244843	-0.794120	-1.397806
71.H	-0.103487	-5.899474	-1.312068
72.H	-4.850708	-3.479225	-1.081745
73.H	2.310725	-3.115644	-1.175594
74.H	1.920750	3.794388	-1.052996
75.H	-3.484955	4.517574	-0.852996
76.H	0.361743	-4.229600	-0.936941
77.H	2.783541	-0.721643	-1.030061
78.H	5.322411	-2.402053	-0.651321
79.H	-0.301073	5.926894	-0.736668
80.H	-5.488716	3.085946	-0.509658
81.H	-2.751639	-0.307595	-0.538506
82.H	-4.359142	-0.997248	-0.337333
83.H	-2.299758	-5.545643	-0.121623
84.H	4.692221	1.272801	-0.165270
85.H	1.385004	2.566963	0.097258
86.H	-4.498172	1.825834	0.244702
87.H	6.563493	-0.329706	0.430626
88.H	3.107381	-3.437196	0.382292
89.H	1.600259	-2.528549	0.332705
90.H	1.349385	4.272527	0.551446
91.H	-2.160033	6.426786	0.741344
92.H	7.026441	1.368468	0.653251

93.H	-4.911989	-4.847935	0.873628
94.H	-4.706318	3.394827	1.052550
95.H	-0.425129	-6.650203	1.046046
96.H	-3.673220	5.610910	1.180358
97.H	5.401376	3.105150	1.342501
98.H	-5.214165	-2.271898	1.334688
99.H	4.950894	-3.138325	1.472564
100.H	0.216464	-5.093928	1.592755
101.H	-3.651633	-5.721995	1.764776
102.H	3.672017	2.762696	1.539818
103.H	0.085168	5.555331	1.863479
104.H	6.624666	0.369172	2.060375
105.H	-1.339466	-5.682675	2.218105
106.H	-2.830767	0.330590	1.906355
107.H	-2.725963	6.454740	2.421174
108.H	6.143491	-2.065362	2.228425
109.H	-3.998871	-1.383860	2.268561
110.H	-5.002922	-4.975251	2.638773
111.H	-3.079973	2.016289	2.307544
112.H	-2.451708	3.980998	2.584680
113.H	4.841487	2.209359	2.762321
114.H	1.265076	2.064327	2.499955
115.H	0.096067	4.014793	2.739350
116.H	0.650044	-2.383515	2.544616
117.H	-2.928021	-3.622512	2.881372
118.H	-5.100552	-2.513429	3.084893
119.H	-0.768447	-3.353453	2.884011
120.H	3.132085	-2.116981	2.879190
121.H	5.293734	-3.274703	3.207450
122.H	-0.689632	5.440916	3.451368
123.H	2.700796	1.362140	3.214997
124.H	-0.881997	2.047202	3.513624
125.H	-2.042185	-1.284472	3.481238
126.H	5.313915	-0.218429	3.956911
127.H	1.385971	-0.631436	3.930555
128.H	-2.028423	0.894515	4.242874
129.H	3.604700	-0.188691	4.422656
130.H	-0.643360	-1.537720	4.554317
131.H	4.610641	-1.586737	4.833987
132.H	0.767541	0.867644	4.666524
133.N	-1.449283	1.647700	0.928994
134.N	-0.961125	-2.054416	1.160687
135.N	1.956543	0.318239	1.472520
136.N	-0.433981	0.026624	3.104942
137.Sb	0.490377	-0.345535	-2.909530
138.Si	-1.169267	-0.686674	-4.887406
139.Si	2.132451	1.326401	-4.037635
140.Si	-1.699714	3.306066	0.329990
141.Si	-2.181644	-3.204691	0.571659
142.Si	3.583087	-0.367128	1.234150
143.Th	-0.030911	-0.084840	0.353761

Energy: −758.34376372 eV

References

1. B. M. Gardner, G. Balázs, M. Scheer, F. Tuna, E. J. L. McInnes, J. McMaster, W. Lewis, A. J. Blake, S. T. Liddle, *Angew. Chem. Int. Ed*. **2014**, 53, 4484.
2. B. M. Gardner, W. Lewis, A. J. Blake, S. T. Liddle, *Organometallics* **2015**, *34*, 2386.
3. E. P. Wildman, G. Balázs, A. J. Wooles, M. Scheer, S. T. Liddle, *Nat.Commun.* **2016**, 7, 12884.
4. C. Marquardt, O. Hegen, M. Hautmann, G. Balázs, M. Bodensteiner, A. V. Virovets, A. Y. Timoshkin, M. Scheer, *Angew. Chem. Int. Ed.* **2015**, *54*, 13122.
5. U. Englich, K. Hassler, K. Ruhlandt-Senge, F. Uhlig, *Inorg. Chem.* **1998**, *37*, 3532.
6. H. H. Karsch, F. Bienlein, T. Rupprich, F. Uhlig, E. Herrmann, M. Scheer, *Synth. Methods Organometallic and Inorg. Chem.*, Vol. 3, Thieme, **1996**, pp. 58-65.
7. C. Fonseca Guerra, J. G. Snijders, G. te Velde E. J. Baerends, *Theor. Chem. Acc.* **1998**, *99*, 391.
8. G. te Velde, F. M. Bickelhaupt, S. J. A. van Gisbergen, C. Fonseca Guerra, E. J. Baerends, J. G. Snijders T. Ziegler, *J. Comput. Chem.* **2001**, *22*, 931.
9. S. H. Vosko, L. Wilk, M. Nusair, *Can. J. Phys.* **1980**, *58*, 1200.
10. A. D. Becke, *Phys. Rev. A.* **1988**, *38*, 3098.
11. J. P. Perdew, *Phys. Rev. B.* **1986**, *33*, 8822.
12. S. Portmann, H. P. Luthi, *Chimia* **2000**, *54*, 766.
13. NBO 5.0: E. D. Glendening, J. K. Badenhoop, A. E. Reed, J. E. Carpenter, J. A. Bohmann, C. M. Morales, F. Weinhold, (Theoretical Chemistry Institute, University of Wisconsin, Madison, WI, 2001); http://www.chem.wisc.edu/~nbo5.
14. R. F. W. Bader, *Atoms in Molecules: A Quantum Theory*, Oxford University Press, New York, 1990.
15. R. F. W. Bader, *J. Phys. Chem. A.* **1998**, *102*, 7314.
16. http://www.quimica.urv.es/XAIM.
